# Chaperone-Usher Pili Loci of Colonization Factor-Negative Human Enterotoxigenic *Escherichia coli*

**DOI:** 10.3389/fcimb.2016.00200

**Published:** 2017-01-06

**Authors:** Felipe Del Canto, Miguel O'Ryan, Mirka Pardo, Alexia Torres, Daniela Gutiérrez, Leandro Cádiz, Raul Valdés, Aquiles Mansilla, Rodrigo Martínez, Daniela Hernández, Benjamin Caro, Myron M. Levine, David A. Rasko, Christopher M. Hill, Mihai Pop, O. Colin Stine, Roberto Vidal

**Affiliations:** ^1^Programa de Microbiología y Micología, Instituto de Ciencias Biomédicas, Facultad de Medicina, Universidad de ChileSantiago, Chile; ^2^Facultad de Química y Biología, Universidad de Santiago de ChileSantiago, Chile; ^3^Center for Vaccine Development, University of Maryland School of MedicineBaltimore, MD, USA; ^4^Department of Microbiology and Immunology, Institute for Genome Sciences, University of Maryland School of MedicineBaltimore, MD, USA; ^5^Center for Bioinformatics and Computational Biology, University of Maryland Institute for Advanced Computer StudiesCollege Park, MD, USA; ^6^Department of Epidemiology and Public Health, University of Maryland School of MedicineBaltimore, MD, USA

**Keywords:** ETEC, adhesin, adhesin negative-ETEC, colonization factors, chaperone-usher pili, genome analysis

## Abstract

Enterotoxigenic *Escherichia coli* (ETEC) is one of the most common causes of diarrhea worldwide. Among the 25 different ETEC adhesins, 22 are known as “colonization factors” (CFs), of which 17 are assembled by the chaperone-usher (CU) mechanism. Currently, there is no preventive therapy against ETEC, and CFs have been proposed as components for vaccine development. However, studies of diarrhea-causing ETEC strains worldwide indicate that between 15 and 50% of these are negative for known CFs, hindering the selection of the most widespread structures and suggesting that unknown adhesins remain to be identified. Here, we report the result of a comprehensive analysis of 35 draft genomes of ETEC strains which do not carry known adhesin genes; our goal was to find new CU pili loci. The phylogenetic profiles and serogroups of these strains were highly diverse, a majority of which produced only the heat-labile toxin. We identified 10 pili loci belonging to CU families β (1 locus), γ_2_ (7 loci), κ (1 locus), and π (1 locus), all of which contained the required number of open reading frames (ORFs) to encode functional structures. Three loci were variants of previously-known clusters, three had been only-partially described, and four are novel loci. Intra-loci genetic variability identified would allow the synthesis of up to 14 different structures. Clusters of putative γ_2_-CU pili were most common (23 strains), followed by putative β-CU pili (12 strains), which have not yet been fully characterized. Overall, our findings significantly increase the number of ETEC adhesion genes associated with human infections.

## Introduction

Enterotoxigenic *Escherichia coli* (ETEC), which includes a wide diversity of strains, are one of six categories of diarrheagenic *E. coli* (Croxen et al., 2013). These ETEC are a common cause of watery diarrhea worldwide, primarily affecting children living in resource-poor settings of developing countries and travelers who visit these endemic regions (Qadri et al., 2005). Humans are the natural reservoir for ETEC; transmission is associated with consumption of food or water contaminated with human feces (Qadri et al., 2005). After infecting the small bowel epithelium, ETEC induce electrolyte and water loss by producing at least one of two enterotoxins, which distinguish them from other diarrheagenic *E. coli*: a heat stable toxin (ST) and/or a heat labile toxin (LT) (Turner et al., 2006). Strains that infect humans can produce two different types of STs, STh (human variant), and STp (pig variant), but only one type of LT (LT-I) (Croxen et al., 2013). An essential part of infection is attachment to intestinal cells, which ETEC accomplish using a diverse array of adhesins; among these, colonization factors (CFs) have been the most studied to date (Croxen et al., 2013).

Currently, no effective preventive therapy against ETEC is available. Vaccines could be a feasible alternative to reduce the associated morbidity and mortality, particularly in resource-poor settings in developing countries (Ahmed et al., 2013). Epidemiological studies, including characterization of ETEC isolates worldwide, have been the basis for selection of the most widespread antigens for vaccine development. Both enterotoxins and ETEC adhesins have been proposed and tested as components for vaccine candidates (Isidean et al., 2011; O'Ryan et al., 2015; Zhang and Sack, 2015). Thus, CFs CFA/I, CS1, CS2, CS3, CS4, CS5, CS6, and CS7, or their components, have been included in several formulations, however none of these candidates have passed phase III trials (O'Ryan et al., 2015; Zhang and Sack, 2015).

Although 22 different CFs and three other non-pili adhesins (Tia, TibA, and EtpA) have been identified in ETEC strains, a key obstacle to building an effective vaccine based on these antigens is the significant proportion (15–50%) apparently lacking known CFs or adhesins (Del Canto et al., 2011; Isidean et al., 2011). These strains may be reflecting our inability to reproduce proper conditions for target expression in the laboratory, or recognition inability by antibodies, probes or primers due to mutations generating closely-related variants. Importantly, negative results may reflect the existence of novel adhesins.

**Figure 1:**
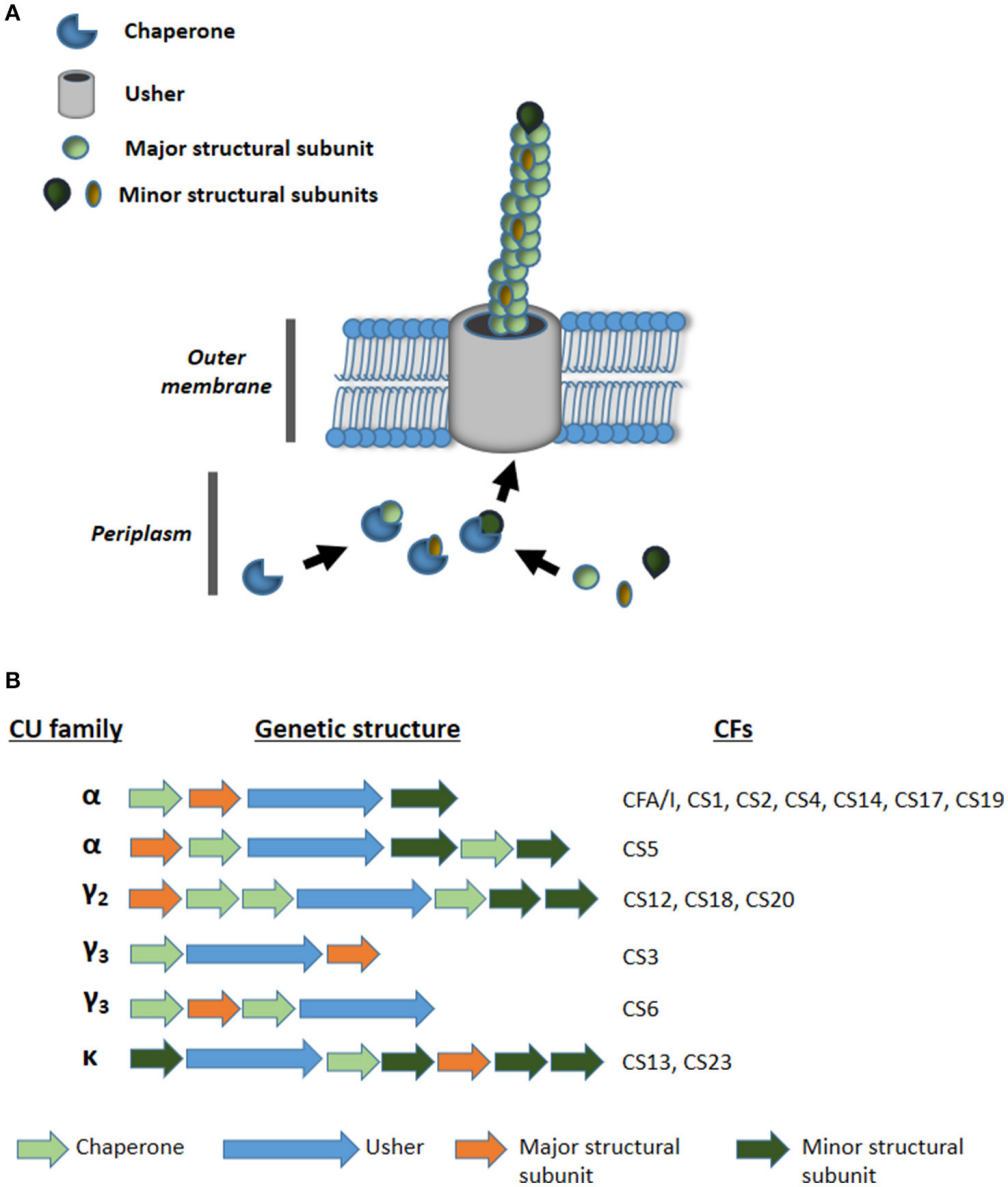
**(A)** Schematic representation of the assembly of chaperone usher (CU) pili. **(B)** Genetic organization of CU pili loci families α, γ_2_, γ_3_, and κ encoding known colonization factors (CFs) of ETEC.

Current advances in massive genome sequencing may address these problems. In this work, our aim was to find novel adhesion loci by analyzing the draft genomes of 35 ETEC strains that appeared negative for currently known adhesins, hereon referred to as AN-ETEC strains. We focused particularly on loci encoding pili assembled by the chaperone-usher pathway (CU pili), which includes most of the currently known CFs (Madhavan and Sakellaris, 2015). These structures are composed of two or more structural subunits, the most abundant of which is the major structural subunit (Figure 1; Waksman and Hultgren, 2009). The remaining are classified as minor subunits, or tip subunits if they constitute the tip of the pilus (Waksman and Hultgren, 2009). The assembly of the pilus structure requires the action of two types of proteins: the usher and the chaperones. The usher is an outer membrane pore forming protein through which structural subunits are exported (Waksman and Hultgren, 2009). The export process is assisted by one or more chaperones that binds and fold structural subunits in the periplasm (Waksman and Hultgren, 2009). The amino acid sequence of the usher is used to classify CU pili, in a phylogenetic context, into six different families: α, β, γ, κ, π, and σ (Nuccio and Bäumler, 2007). Since the γ CU-family is the most numerous, it has been divided into four subfamilies: γ_1_, γ_2_, γ_3_, and γ_4_ (Nuccio and Bäumler, 2007). Fifteen of the 22 ETEC CFs have CU pili assigned to one of these families. Thus, CFA/I, CS1, CS2, CS4, CS5, CS14, CS17, and CS19 belong to the α family; CS12, CS18, and CS20 belong to the γ_2_ family; CS3 and CS6 belong to the γ_3_ family; and CS13 and CS23 belong to the κ family (Madhavan and Sakellaris, 2015; Figure 1). CS7, CS15, and C22 have also been considered CU pili, as they share similarity with known structures from families α (CS7 is similar to CS5) and γ_3_ (CS15 and CS22 are similar to SEF14 produced by *Salmonella enterica* serovar Enteritidis; Pichel et al., 2000; Del Canto et al., 2012). However, they have not been assigned to any particular family (Madhavan and Sakellaris, 2015). Consequently, as most of the CFs are CU pili, we expected to find novel CU pili loci, or genetic variants of those already known, in genomes of ETEC strains which lacks CFs.

## Materials and methods

### Strains

Thirty five ETEC strains were included in the study, each of which were considered negative for known adhesins (AN-ETEC), because no product was obtained in PCR analyses aimed at detecting loci encoding CFA/I, CS1, CS2, CS3, CS4, CS5, CS6, CS7, CS8, CS12, CS13, CS14, CS15, CS17, CS18, CS19, CS20, CS21, CS22, CS23, Tia, TibA, or EtpA. The complete list of primers used in this work is included in Table S2 (Supporting Information). All AN-ETEC strains had been isolated from children under five years of age with watery diarrhea: 14 of the 35 strains were obtained in Chile and had been previously characterized according to O serogroup and enterotoxin genes (Del Canto et al., 2011). The other 21 isolates were obtained as part of the Global Enterics Multicenter Study (GEMS) in Kenya, Mali, Mozambique, The Gambia, Pakistan, India, and Bangladesh (Kotloff et al., 2013). Enterotoxin gene repertoires were determined using standard procedures as described previously by our research group (Del Canto et al., 2011; Panchalingam et al., 2012). For this group of strains (GEMS' strains), serotyping of the O antigen was carried out by seroagglutination at the Programa de Microbiología y Micología of the Universidad de Chile.

### Sequencing

To obtain draft genomic sequences for the 35 AN-ETEC strains, genomic DNA was purified using the Wizard Genomic DNA purification kit (Promega), processed according to the Illumina Paired End Protocol with inserts of 400 bp, and sequenced in a HiSeq 2000 platform at the Institute for Genome Sciences (Baltimore, MD). Reads were analyzed using FastQC v0.10.1 (Babraham Bioinformatics, Babraham Institute, Cambridge, UK. Available: http://www.bioinformatics.bbsrc.ac.uk/projects/fastqc/) and assembled using MaSuRCA 2.2.1 or SPAdes 3.1.0 (Bankevich et al., 2012; Zimin et al., 2013). The best of the two assemblies was finally chosen. Best kmer length for assembly was predicted with KmerGenie v.16476 prior to assembly with SPAdes (Chikhi and Medvedev, 2014). Assembly statistics were obtained with Quast v2.3 and the completeness/contamination report with CheckM (Gurevich et al., 2013; Parks et al., 2015). Sequence annotation was first performed using the Rapid Annotation Subsystem Technology (RAST; Aziz et al., 2008) and then through the NCBI Prokaryotic Genome Annotation Pipeline (National Center for Biotechnology Information, Bethesda MD, USA. Available: http://www.ncbi.nlm.nih.gov/genome/annotation_prok/).

### Phylogeny

A phylogenetic tree was inferred based on single nucleotide polymorphisms in draft genomes of our AN-ETEC, by using the CSI phylogeny 1.1, a tool on the Center for Genomic Epidemiology server (Kaas et al., 2014). The following genomic sequences, containing their respective plasmids, were included in the analysis: *E*. *coli* B, *E. coli* K-12 MG1655, *E. coli* HS, *E. coli* W, *E. coli* ED1a, *E. coli* IAI-1, *E. coli* SE11, *E. coli* SE15 and ETEC prototype strains H10407, E24377A, and B7A. The accession numbers of these sequences are included in Table S3 of the Supporting Information. The genomic sequence of *E. coli* K-12 MG1655 was set as the reference. A total of 2,481,579 positions were found in all analyzed genomes (53.46% of the reference). The tree was drawn using Mega 6.06 (Tamura et al., 2013). Phylogenetic groups were assigned according to the PCR protocol described by Clermont et al. and complemented with manual analysis of the target genes (Clermont et al., 2013). Sequence types were assigned using the MLST 1.7 of the CGE, according to the scheme proposed by Wirth et al. based on genes *adk, fumC, gyrB, icd, mdh, purA*, and *recA* (Wirth et al., 2006; Larsen et al., 2012).

### Loci identification and analysis

Putative adhesin loci in AN-ETEC genomes were identified first by screening of major CF subunits and then by screening of usher genes using Blast Ring Image Generator (BRIG) v0.95 and/or Large-scale Blast Score Ratio (LS-BSR) (Alikhan et al., 2011; Sahl et al., 2014). In LS-BSR, a blast score ratio (BSR) between 0.00 and 1.00 was obtained for every screened gene in an individual genome. BSR values of 1.00 indicate the maximum sequence identity. For CF screening, genes encoding the following structural subunits were considered: CFA/I, CS1, CS2, CS3, CS4, CS5, CS6, CS7, CS8, CS12, CS13, CS14, CS15, CS17, CS18, CS19, CS20, CS21, CS22, and CS23. For the usher screening, a set of 162 genes of CU pili belonging to the α (16 genes), β (5 genes), γ_1_ (25 genes), γ_2_ (7 genes), γ_3_ (15 genes), γ_4_ (40 genes), κ (9 genes), π (25 genes), and σ families (20 genes), were selected based primarily on a previous review (Nuccio and Bäumler, 2007). The complete list of genes used for screening is included in Table S1 (supporting information). Homolog genes were located within AN-ETEC genomes and their genetic context was analyzed to look for open reading frames (ORFs) encoding putative structural subunits, chaperones, transcriptional regulators, and others that could be part of a CU pili locus. Gene clusters were characterized and compared to known CU pili loci using the NCBI ORF finder (National Center for Biotechnology Information, Bethesda MD, USA. Available: http://www.ncbi.nlm.nih.gov/projects/gorf/), Clustal Omega and/or Unipro UGENE (Sievers et al., 2011; Okonechnikov et al., 2012). In most cases, phylogenetic trees were inferred according to amino acid sequence alignment using Mega 6 (Tamura et al., 2013). Heat maps for screening with LS-BSR or percentage of identity were draw with the Multiple experiment Viewer (MeV) v4.9.0, and loci comparison graphics were drawn with EasyFig v2.1 (Saeed et al., 2006; Sullivan et al., 2011).

### Data availability

The 35 AN-ETEC genomes were partially sequenced as part of an ETEC whole genome shotgun project and deposited in GenBank under the accession BioProject PRJNA287625. In addition, a file containing the amino acid sequences of the major structural subunits was included as Supplementary Material (Supplementary Data Sheet 1). Chilean bacterial isolates may be requested by contacting corresponding authors and GEMS isolates may be requested at http://www.medschool.umaryland.edu/GEMS/GEMS-Data--Specimen-Requests/.

## Results

**Figure 2:**
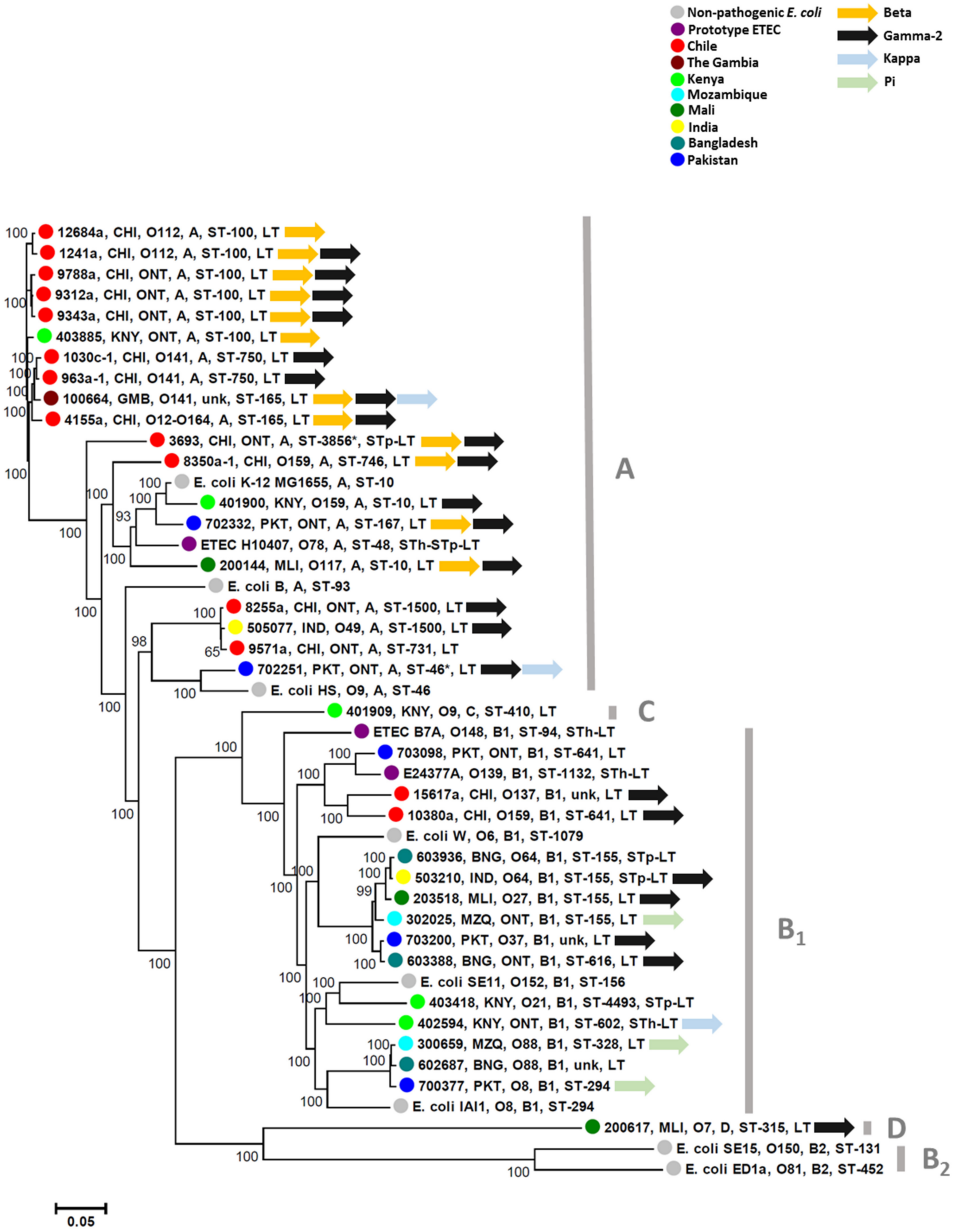
**Phylogenetic diversity of ETEC strains included in this study**. Tree depicting genetic relationships among AN-ETEC strains based on a single nucleotide polymorphism-calling procedure (CSI phylogeny). Font colors indicate different geographical origins (countries). In each node, the indicated features are as follows: strain code, country of origin, O serogroup, phylogroup, sequence type, and enterotoxins. The following non-pathogenic strains (in black fonts) and ETEC prototype strains (in purple fonts) were included in the analysis: *E*. *coli* B (REL606), *E. coli* W (Crooks), *E. coli* K-12 MG1655, *E. coli* HS, *E. coli* ED1a, *E. coli* IAI1, *E. coli* SE11, *E. coli* SE15, ETEC H10407, ETEC E24377A, and ETEC B7A. Asterisks in ETEC 3693 and ETEC 702251 indicate sequence types suggested by the MLST 1.7 tool when non-perfect matching occurred. Unk, unknown sequence type.

Thirty-five AN-ETEC strains genomes were sequenced in order to identify putative adhesin loci. These strains belonged to 13 different somatic serogroups (O serogroup) of which 10 were non-typeable (ONT), and 82% (29/35) were positive for LT only (lacking ST). Sequencing and assembly statistics are shown in Table 1. Sequencing coverage ranged between 75X and 353X, and the average length of the assembled draft genomes was 5,126,274 bp, which was close to the expected size for a pathogenic *E. coli* strain (Lukjancenko et al., 2010). Estimates of completeness and contamination for our draft genomes, assessed using a set of *Enterobacteriaceae*-specific markers, indicated that the vast majority of them were nearly complete (≥95%) and had low levels of contamination (≤5%). Only for two strains, ETEC 1241a and ETEC 4155a, results suggest medium contamination levels (6.04 and 5.04%, respectively). Phylogenetic characterization of our AN-ETEC strains, according to their genomes by SNP calling and *in silico* multiple locus sequence typing (MLST), was generally consistent with phylogenetic groups and serogroups, rather than with the geographical origin of the strains. Three main groups of strains can be identified in the tree shown in Figure 2. One group included 10 AN-ETEC strains primarily isolated in Chile, belonging to phylogroup A and sequence types ST-100, ST-750, and ST-165. The second group included 10 AN-ETEC strains belonging also to the phylogroup A, but associated with eight different sequence types. Non-pathogenic strains *E. coli* B, *E. coli* K-12 MG1655, *E. coli* HS, and the prototype ETEC H10407, all known members of the phylogroup A (Wurpel et al., 2013), were located in this group. The third group contained 14 strains belonging to the phylogroup B_1_. This group was associated with eight different sequence types, none of which are present in phylogroup A AN-ETEC, including known non-pathogenic *E. coli* strains, *E. coli* W, *E. coli* SE11, *E. coli* IAI1, and the prototypic ETEC strains ETEC E24377A and ETEC B7A (Wurpel et al., 2013). Only one of our AN-ETEC (ETEC 200617) belonged to the phylogroup D. Overall, the AN-ETEC collection analyzed in this work is genetically diverse belonging mainly to phylogroups A and B_1_. No widely distributed or predominant serogroup or sequence type was identified.

**Table 1:** **Summary of draft genomic sequences of ETEC strains obtained in this work**.

**Strain**	**Origin**	Coverage^*^	**Assembler**	**Total length (bp)**	**Contigs**	**N50**	**% GC**	**% Completeness^†^**	**% Contamination^†^**	**GenBank accession code**
3693	Chile	226	SPAdes	5,001,931	251	75,875	50.6	99.96	0.40	LGLX00000000
12684a	Chile	182	MaSuRCA	4,887,648	259	84,003	50.9	95.33	2.56	LGLY00000000
15617a	Chile	149	MaSuRCA	4,979,396	149	133,333	50.8	96.63	2.50	LGLZ00000000
963a-1	Chile	144	MaSuRCA	5,000,982	193	108,211	50.5	92.44	3.20	LGMA00000000
1030c-1	Chile	157	MaSuRCA	5,318,135	203	86,519	50.6	99.65	2.80	LGMB00000000
1241a	Chile	179	MaSuRCA	5,498,099	250	102,427	50.8	100	6.04	LGMC00000000
4155a	Chile	175	MaSuRCA	5,052,165	171	104,482	50.6	97.58	5.04	LGMD00000000
8255a	Chile	213	MaSuRCA	5,029,983	289	81,830	50.6	99.65	2.30	LGME00000000
8350a	Chile	170	MaSuRCA	5,024,877	136	138,088	50.7	99.34	0.29	LGMF00000000
9312a	Chile	220	SPAdes	4,940,375	158	150,786	50.6	100	0.08	LGMG00000000
9343a	Chile	120	MaSuRCA	5,049,524	146	151,450	50.7	100	1.22	LGMH00000000
9571a	Chile	207	MaSuRCA	5,119,006	233	137,783	50.7	99.96	2.07	LGMI00000000
9788a	Chile	172	MaSuRCA	4,954,530	130	134,873	50.7	99.73	0.69	LGMJ00000000
10380a	Chile	187	MaSuRCA	4,825,041	147	117,204	50.6	99.96	1.62	LGMK00000000
700377	Pakistan	187	MaSuRCA	5,509,751	282	89,754	50.4	98.06	2.74	LGML00000000
300659	Mozambique	146	MaSuRCA	5,193,583	176	116,939	50.6	99.96	2.37	LGMM00000000
200144	Mali	127	MaSuRCA	5,096,812	211	75,533	50.8	99.96	1.70	LGMN00000000
200617	Mali	168	MaSuRCA	5,474,100	183	177,338	50.3	99.66	1.28	LGMO00000000
503210	India	75	MaSuRCA	5,170,803	119	134,854	50.7	99.96	2.25	LGMP00000000
401909	Kenya	75	MaSuRCA	5,018,235	110	133,005	50.5	99.96	1.19	LGMQ00000000
401900	Kenya	343	SPAdes	4,737,288	139	133,706	50.6	99.96	0.11	LGMR00000000
100664	The Gambia	103	MaSuRCA	5,332,273	201	80,008	50.4	99.96	4.66	LGMS00000000
302025	Mozambique	127	MaSuRCA	5,152,571	172	114,889	50.6	99.96	2.36	LGMT00000000
602687	Bangladesh	109	MaSuRCA	5,029,988	242	67,774	50.8	99.39	2.42	LGMU00000000
402594	Kenya	166	MaSuRCA	5,280,101	173	94,074	50.5	99.96	2.80	LGMV00000000
702251	Pakistan	145	MaSuRCA	5,323,290	334	55,624	50.4	99.34	3.56	LGMW00000000
702332	Pakistan	102	MaSuRCA	5,013,771	167	88,063	50.8	99.96	2.49	LGMX00000000
703098	Pakistan	353	SPAdes	5,356,213	300	168,561	50.6	99.96	0.15	LGMY00000000
603388	Bangladesh	126	MaSuRCA	5,166,599	160	162,966	50.6	99.96	2.85	LGMZ00000000
203518	Mali	165	MaSuRCA	5,036,223	137	130,574	50.8	99.96	1.50	LGNA00000000
403418	Kenya	169	MaSuRCA	5,069,930	135	119,990	50.8	99.96	1.84	LGNB00000000
703200	Pakistan	78	MaSuRCA	5,249,204	128	124,962	50.4	96.26	3.72	LGNC00000000
603936	Bangladesh	150	MaSuRCA	5,224,195	138	120,925	50.7	99.96	1.88	LGND00000000
505077	India	131	MaSuRCA	5,314,101	194	125,041	50.6	98.73	4.55	LGNE00000000
403885	Kenya	131	MaSuRCA	4,988,884	134	121,380	50.9	100	0.47	LGNF00000000

* *Coverage estimated according to the ETEC H10407 genome length, including plasmids*.

† *Estimated by presence of lineage markers using CheckM*.

### CFs-bioinformatical screening

**Figure 3:**
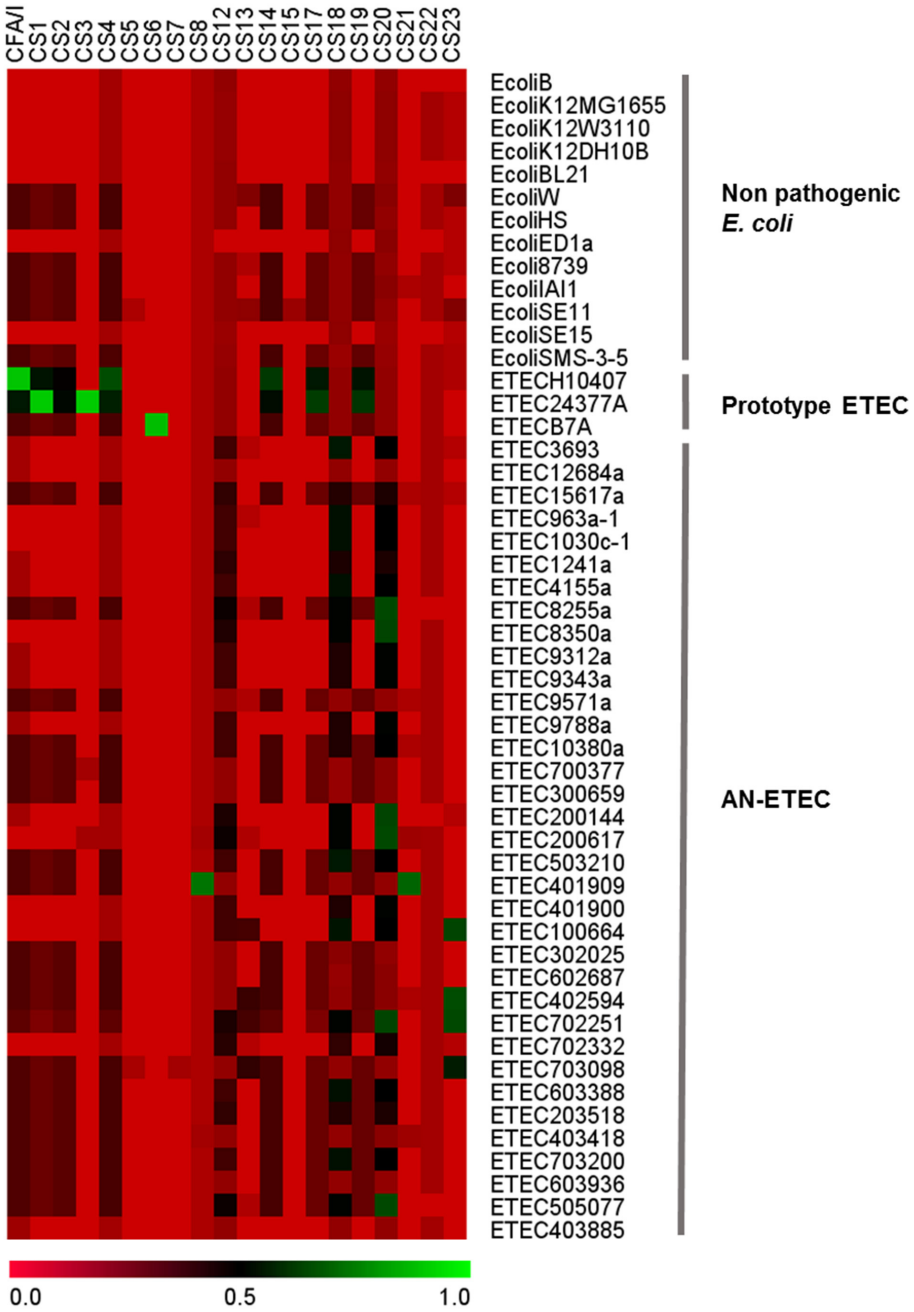
**Bioinformatical screening of colonization factors**. Heat map derived from the screening of CF-structural subunit genes with large scale-blast score ratio (LS-BSR). Genomes of non-pathogenic and ETEC prototype strains were included in the analysis.

Strains in this study had been previously characterized by PCR and all of them had tested negative for known ETEC CFs genes. We used the same genes used in the PCR screening to probe the draft genomes using LS-BSR with the tblastn algorithm (Sahl et al., 2014). As seen in Figure 3, CF genes were not detected in non-pathogenic *E. coli* strains, with blast-score ratio (BSR) values ranging between 0.00 and 0.32. In contrast, they were correctly detected in ETEC prototype strains H10407 (CFA/I), ETEC E24377A (CS1 and CS3), and ETEC B7A (CS6) with BSR values ranging between 0.97 and 1.00. Although most of these AN-ETEC genomes were confirmed negative for known CF genes by this second screening method, the blast-score ratio (BSR) values obtained in some cases (between 0.57 and 0.79) for genes encoding CS8/CS21 (ETEC 401909), CS20 (ETEC 8255a, ETEC 8350a-1, ETEC200144, ETEC200144, ETEC 200617, ETEC 702251 and ETEC 505077), and CS23 (ETEC 100664, ETEC 402594, ETEC 702251 and ETEC 703098), suggest the presence of homologs to those genes.

CS8 and CS21 are homologous structures belonging to the type IV pili family (Madhavan and Sakellaris, 2015). Interestingly, a gene homologous to *cofA* and *lngA*, encoding CS8 and CS21 pilins, respectively, was localized in the ETEC 401909 strain (Kenya, O9, phylogroup C, ST-410, LT), the only AN-ETEC strain with relatively high BSR values (0.79 for CS8 and 0.75 for CS21). Analysis of the PCR primers' target sequences for *cofA* and *lngA* showed discrepancies that may explain negative PCR results (Figure S1A in Supporting Information). The full-length gene nucleotide sequence shared 77% identity with *cofA* and 60% identity with *lngA*, but was 100% identical with the recently discovered CS8b variant, which has yet to be functionally characterized (Njoroge et al., 2015). The alignment of the derived amino acid sequences allowed us to probe this identity (Figure S1B). Therefore, even though the ETEC 401909 strain was negative for detection of *cofA* and *lngA*, it harbored a recently discovered and related variant.

### CU pili

**Figure 4:**
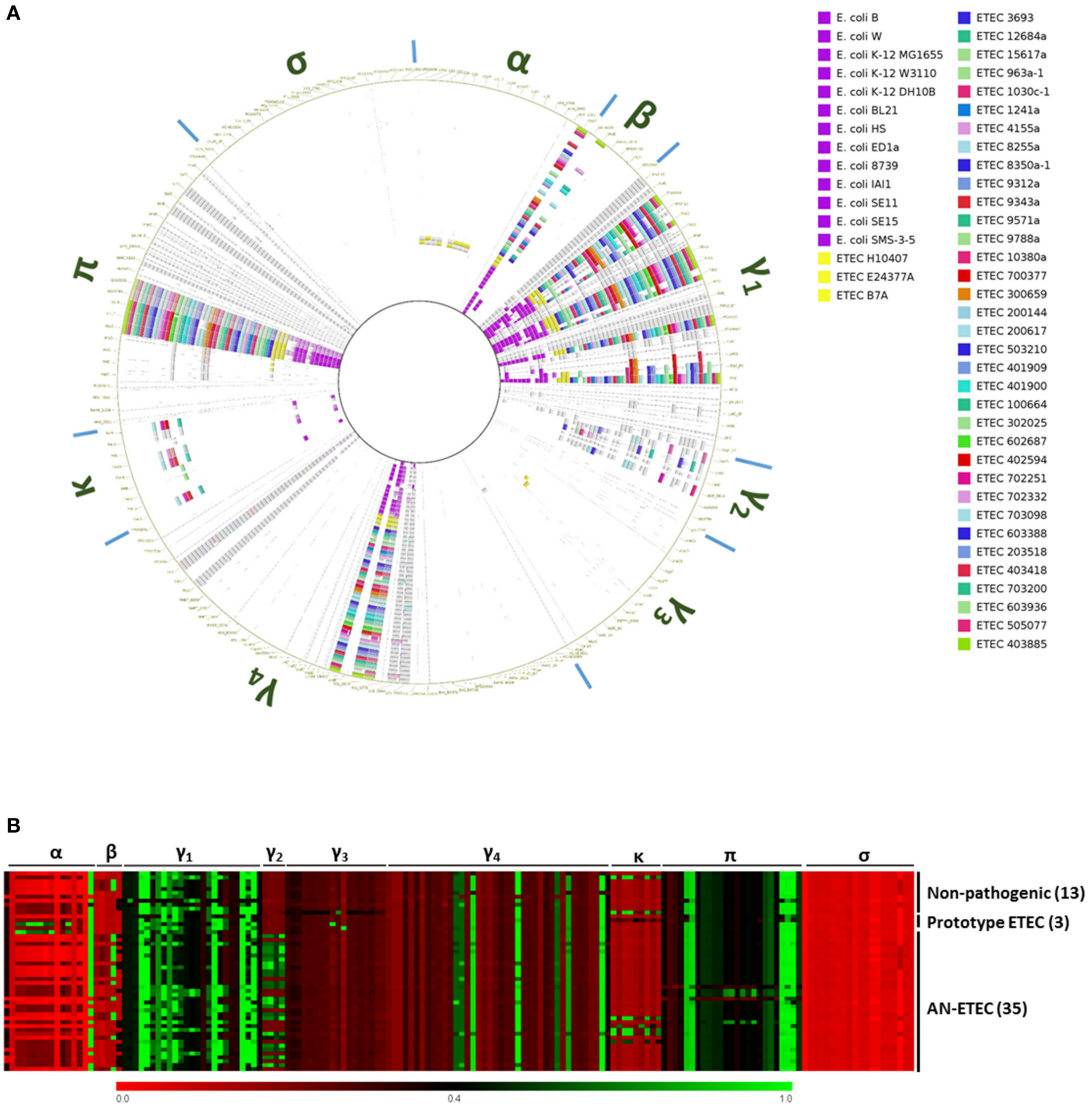
**Bioinformatical screening for chaperone-usher (CU) pili loci among AN-ETEC strains**. **(A)** Screening of usher genes using blastn in blast ring image generator (BRIG). Cut-off values were 70% for identity percentage and 0.01 for the blast *E*-value. Greek letters around the ring indicate columns corresponding to the different CU-pili families. **(B)** Heat map derived from the screening of the same set of usher genes using tblastn in LS-BSR. Columns corresponding to the different CU-pili families are indicated at the top of the map. In **(A,B)** genomes of non-pathogenic *E. coli* strains and ETEC prototype strains were included.

Most of the CFs are CU pili. By screening of the CF structural subunit genes that were targets for PCR detection in AN-ETEC strains, we were able to identify sequences with high similarity (Figure 3). In order to complement this analysis and look for additional potential loci homologous to known CU pili loci, we bioinformatically screened a set of 162 usher genes from AN-ETEC genomes, using the blast- ring image generator (BRIG) with the blastn option and LS-BSR with tblastn (Alikhan et al., 2011; Sahl et al., 2014). Usher genes were chosen because they are present only in a single copy within the CU loci. This analysis included the genomes of 13 non-pathogenic *E. coli* strains and three ETEC prototype strains. As shown in Figure 4, the results derived from both approaches were similar. As previously reported, there is a set of core CU pili loci in *E. coli* including *mat* (α family), *fim* (γ_1_), *yde* (γ_1_), *yad* (γ_4_), *yeh* (γ_4_), *yfc* (π), and *ybg* (π) (Wurpel et al., 2013). Usher genes from all of these loci were detected in both non-pathogenic and pathogenic ETEC genomes. Usher genes from loci encoding known CFs of ETEC prototype strains were detected, namely CFA/I of ETEC H10407 and CS1 of ETEC E24377A in the α family, along with CS3 of ETEC E24377A and CS6 of ETEC B7A in the γ_3_ family. Consistent with CF gene screening, homologous sequences encoding ushers from the γ_2_ and κ families were found in several AN-ETEC strains but were not detected in the majority of the non-pathogenic strains. Similar results were observed for usher genes from the β family and from some representatives of the π family. No homologs of usher genes from the σ family were detected in our set of AN-ETEC genomes. Our results suggest that AN-ETEC strains may contain CU-pili loci encoding structures from the β, γ_2_, κ, and π families.

### Kappa CU pili family

**Figure 5:**
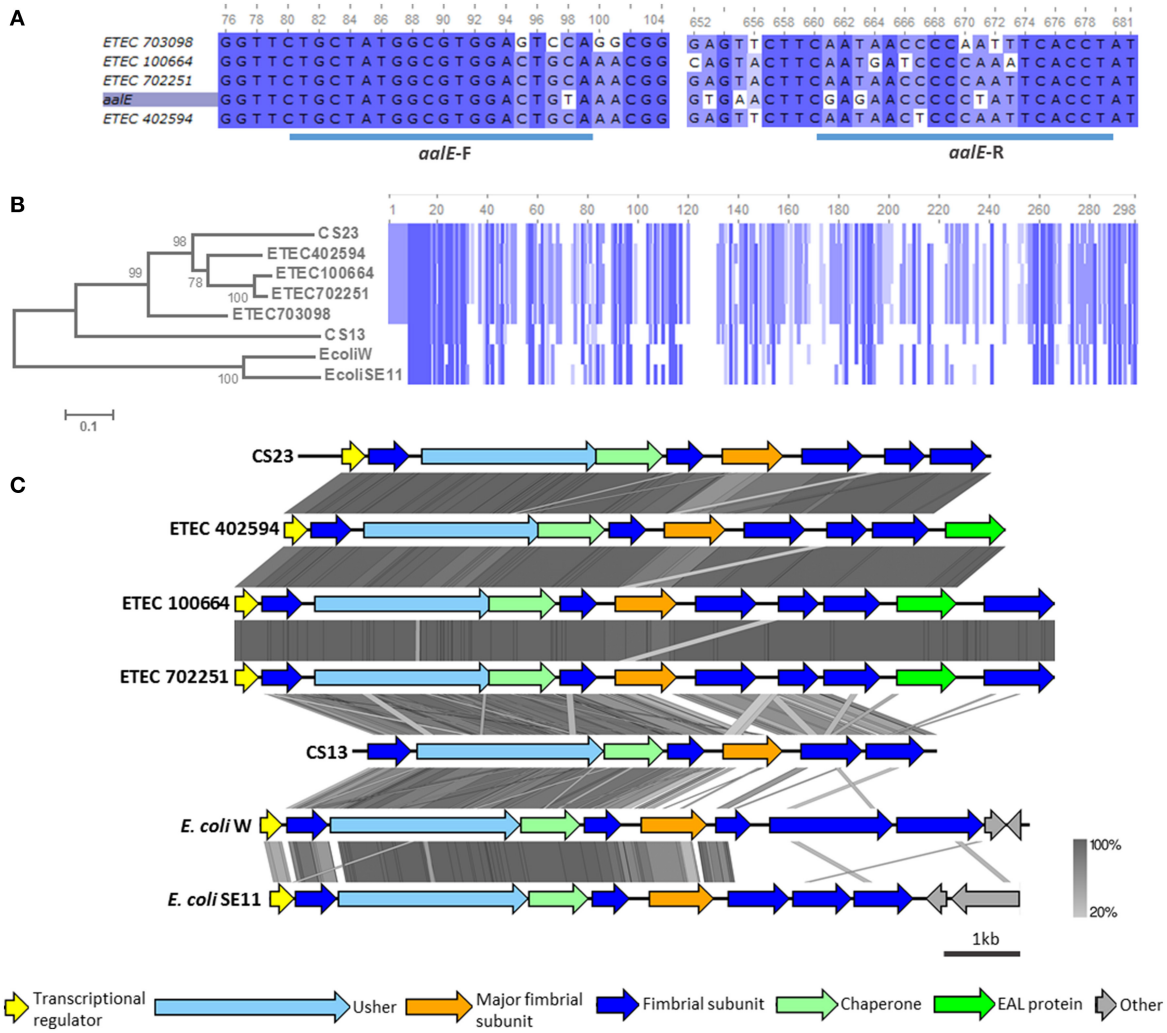
**Analysis of κ-CU pili loci found in AN-ETEC strains**. **(A)** Alignment of the gene encoding CS23 major subunit (*aalE*) with homologs found in AN-ETEC strains. The blue bars at the bottom indicate the primer's target sequences of forward and reverse primers. **(B)** Alignment of the amino acid sequences of AalE and the homologs found in AN-ETEC strains. Putative major subunits of κ-CU pili of non-pathogenic strains *E. coli* W and *E. coli* SE11 were included. Blue blocks indicate identical regions and the color fades as the identity percentage decreases. The tree was inferred from alignment using the neighbor joining method with 1000 bootstrap replicates. **(C)** Alignment of predicted κ-CU pili loci found in AN-ETEC strains using tblastx in Easyfig. Loci encoding CS23 and CS13, such as those κ-CU pili loci found in *E. coli* W and *E. coli* SE11, were included in the analysis.

According to our CF gene screening, genes homologous to *aalE*, which encodes the CS23 major subunit, would be harbored by four AN-ETEC strains (and were not restricted to any particular phylogroup or ST): ETEC 100664, ETEC 402594, ETEC 702251, and ETEC 703098. This observation was consistent with the fact that sequences homologous to usher genes belonging to the kappa family were detected in these strains (BSR values between 0.41 and 0.99, Figure 4B). However, the *aalE* primer did not match perfectly in any of these genomes, which explains the negative PCR results (Figure 5A). Based on the organization of the genetic cluster encoding CS23 and CS13 (Nuccio and Bäumler, 2007; Madhavan and Sakellaris, 2015), complete loci were identified in three of the four mentioned AN-ETEC strains (ETEC 100664, ETEC 402594, and ETEC 702251). This includes six genes encoding structural subunits, one for a chaperone, and one for the usher (Nuccio and Bäumler, 2007; Madhavan and Sakellaris, 2015). In the case of the ETEC 703098 strain, the sequencing did not cover the complete cluster and only five structural subunits were found: the chaperone and the 3′ half portion of the usher gene. Alignment of the amino acid sequences of the putative major structural subunits (PMSs) indicated that these identified loci would encode pili more closely-related to CS23 than to CS13 (Figure 5B). Identity percentages between AalE and the novel PMSs were 68% for ETEC 402594, 67% for ETEC 100664 and ETEC 702251, and 59% for ETEC 703098. As κ-CU pili loci are also present in the genomes of non-pathogenic *E. coli* W and *E. coli* SE11, we included these in the analysis. However, the κ-PMS sequences derived from both strains are different from those found in AN-ETEC strains, sharing only 26 and 25% amino acid identity with AalE (Figure 5B). The regions of identity among all the κ-PMSs resided mainly in the first 30 amino acids, which corresponds with the predicted AalE signal sequence for secretion to the periplasm (Del Canto et al., 2012).

Analysis of the κ-CU pili loci of AN-ETEC strains showed that they share the same genetic organization (Figure 5C). A gene encoding a putative transcriptional regulator, similar to that harbored in the CS23 locus, was found in the three AN-ETEC strains having a complete κ-CU pili locus. In addition, a putative seventh structural subunit gene was found in the ETEC 100664 and ETEC 702251 κ-CU pili loci, in which the contig length allowed for recognition of downstream sequences. This was located downstream a gene encoding a putative phosphodiesterase (EAL domain-containing protein). Alignment using tblastx indicated that κ-CU pili loci carried by ETEC 100664 and ETEC 702251 are nearly identical, and differ from loci harbored by ETEC 402594 and the *aal* locus, mainly in the PMS sequence. A higher level of dissimilarity for all ORFs was observed between the κ-CU pili loci of AN-ETEC strains, the *csh* locus (CS13), and κ-CU pili loci of *E. coli* W and *E. coli* SE11.

Screening of usher genes also allowed us to identify a locus similar to the κ-CU pili loci, encoding fimbriae AF/R1 of rabbit enteric pathogen *E. coli* RDEC-1 and F18 of porcine ETEC in the AN-ETEC 602687 strain. However, as sequencing did not allow for assembly of a complete locus, we did not analyze this cluster.

### Gamma-2 CU pili family

**Figure 6:**
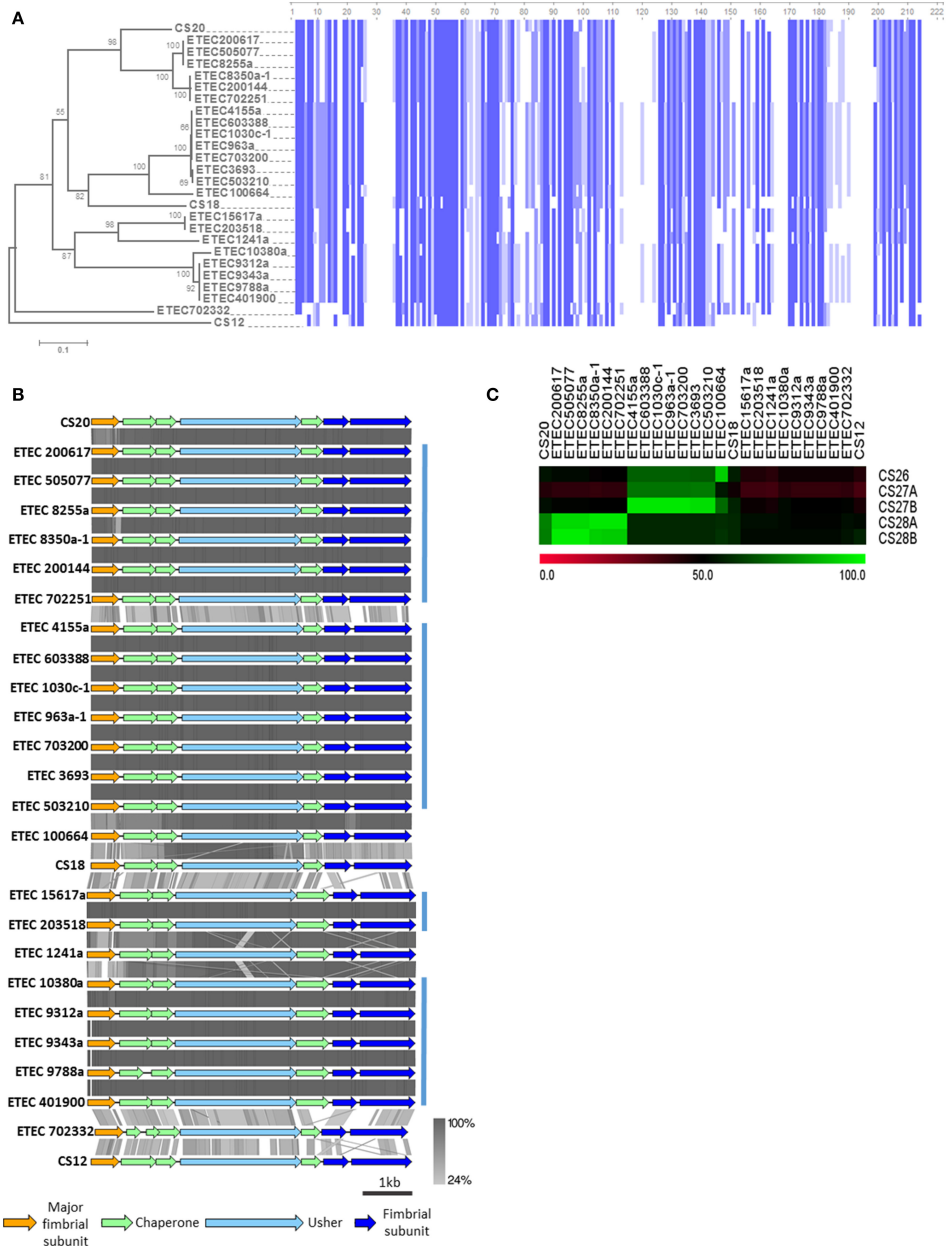
**Analysis of γ_2_-CU pili loci found in AN-ETEC strains. (A)** Alignment of the amino acid sequences of CS12, CS18, and CS20 major structural subunits, and the homologs found in AN-ETEC strains. Blue blocks indicate identical regions and the color fades as the identity percentage decreases. The tree was inferred from alignment using the neighbor joining method with 1000 bootstrap replicates. **(B)** Alignment of the predicted γ_2_-CU pili loci found in AN-ETEC strains using tblastx in Easyfig. Loci encoding CS12, CS18, and CS20 were included. Blue bars at the right indicate groups of loci sharing high identity percentages. **(C)** Identity based-heap map derived from the alignment of nucleotide sequences of CS26, CS27a, CS27b, CS28a, and CS28b with γ_2_-CU pili putative major subunit genes found in AN-ETEC strains. Colors represent percent of identity.

Genomes of six AN-ETEC strains showed high BSR scores for a gene encoding the major structural subunit of CS20, suggesting the presence of γ_2_-CU pili loci in AN-ETEC strains (Figure 3). After complementing this information with the results from our usher genes screening, γ_2_-CU pili loci were identified in 23 AN-ETEC genomes (BSR values ranging between 0.46 and 1.00, Figure 4B). According to the genetic organization of loci encoding γ_2_-CFs CS12, CS18, and CS20, the loci identified in AN-ETEC strains do have the minimum number of genes required to direct synthesis of a functional structure, including three encoding structural subunits, three for chaperones, and one for an usher (Nuccio and Bäumler, 2007; Madhavan and Sakellaris, 2015). However, alignment of amino acid sequences of the PMSs proved that none were identical to the currently known major structural subunits CswA (CS12), FotA (CS18), or CsnA (CS20) and that most of these were closely related to the last two (Figure 6A; Nuccio and Bäumler, 2007; Madhavan and Sakellaris, 2015). Overall, there were six groups derived from the alignment, containing between two and five 100% identical PMS sequences. Putative γ_2_-PMS from two of these groups (PMS harbored by ETEC 200617, ETEC 505707, ETEC 8255a and ETEC 200144, ETEC 702251) plus that harbored by ETEC 8350a-1, shared about 76% identity with CsnA. Five of these six strains belong to the phylogroup A and one to the phylogroup D, representing four different serogroups and five sequence types.

Eight AN-ETEC strains (the group of ETEC 4155a, ETEC 603388, ETEC 1030c-1, ETEC 963a-1, ETEC 703200, the group of ETEC 3693, ETEC 503210, plus ETEC 100664) harbor γ_2_-PMS with 58–59% identity to FotA (Figure 6A). This group includes five phylogroup A strains and three phylogroup B_1_ strains. Other five strains (ETEC 10380a and the group of ETEC 9312a, ETEC 9343a, ETEC 9788a, ETEC 401900) harbor γ_2_-PMS that share 59% identity with CsnA. Four of these strains belong to phylogroup A and three are closely related to ST-100 strains isolated in Chile. Within the other four AN-ETEC strains carrying γ_2_-CU pili loci, there are two identical PMS sharing 51% identity with CsnA (ETEC 1517a and ETEC 203518) and two different sequences with 50 and 49% identity to CsnA (ETEC 1241a and ETEC 702332). Alignment revealed a block of eight amino acids conserved across CS20 and all the γ_2_-PMS of the AN-ETEC strains between positions 50 and 57 (Figure 6A). This region corresponds to the residues 18–25 of the mature CsnA protein (Valvatne et al., 1996). Other 33 conserved residues among all the γ_2_-PMS and the known γ_2_-CFs major subunits were found distributed along the whole extension of the sequences and including single residues, as stretches ranging between two and four amino acids (Figure 6A).

Analysis of the genetic structure and alignment of the complete γ_2_-CU pili loci using tblastx showed that the structure is maintained across AN-ETEC strains and those identity-percentages are consistent with groups described based on γ_2_-PMS sequences (Figure 6B). Differences between CS20 loci and those γ_2_-CU pili loci included in the first group of six AN-ETEC strains resided mainly in the PMS, while, in the rest of the γ_2_-CU pili, it also depends on the putative chaperones, minor structural subunits and even usher genes.

We aligned sequences with those of five, previously described, partial γ_2_-PMS gene sequences: CS26, CS27A, CS27B, CS28A, and CS28B (Nada et al., 2011), using blastn, and found both high similarities and identities. The gamma-2 PMS gene found in ETEC 100664 shared 91% identity with CS26 (Figure 6C). CS27A did not share high identity (≥90%) with any gene sequences in our study. In contrast, a group of seven AN-ETEC strains harbored γ_2_-PMS genes containing sequences highly similar (94–96%) to CS27B. This group also includes strains whose γ_2_-PMS shared 58–59% identity with FotA. Three AN-ETEC bear γ_2_-PMS genes sharing high identity with CS28A (96% identity), as was other three AN-ETEC bear γ_2_-PMS genes almost identical to CS28B with (97% identity). Together, these results suggest that γ_2_-CU pili, other than CS12, CS18, and CS20, might be part of the ETEC adhesin repertoire.

### Pi CU family

**Figure 7:**
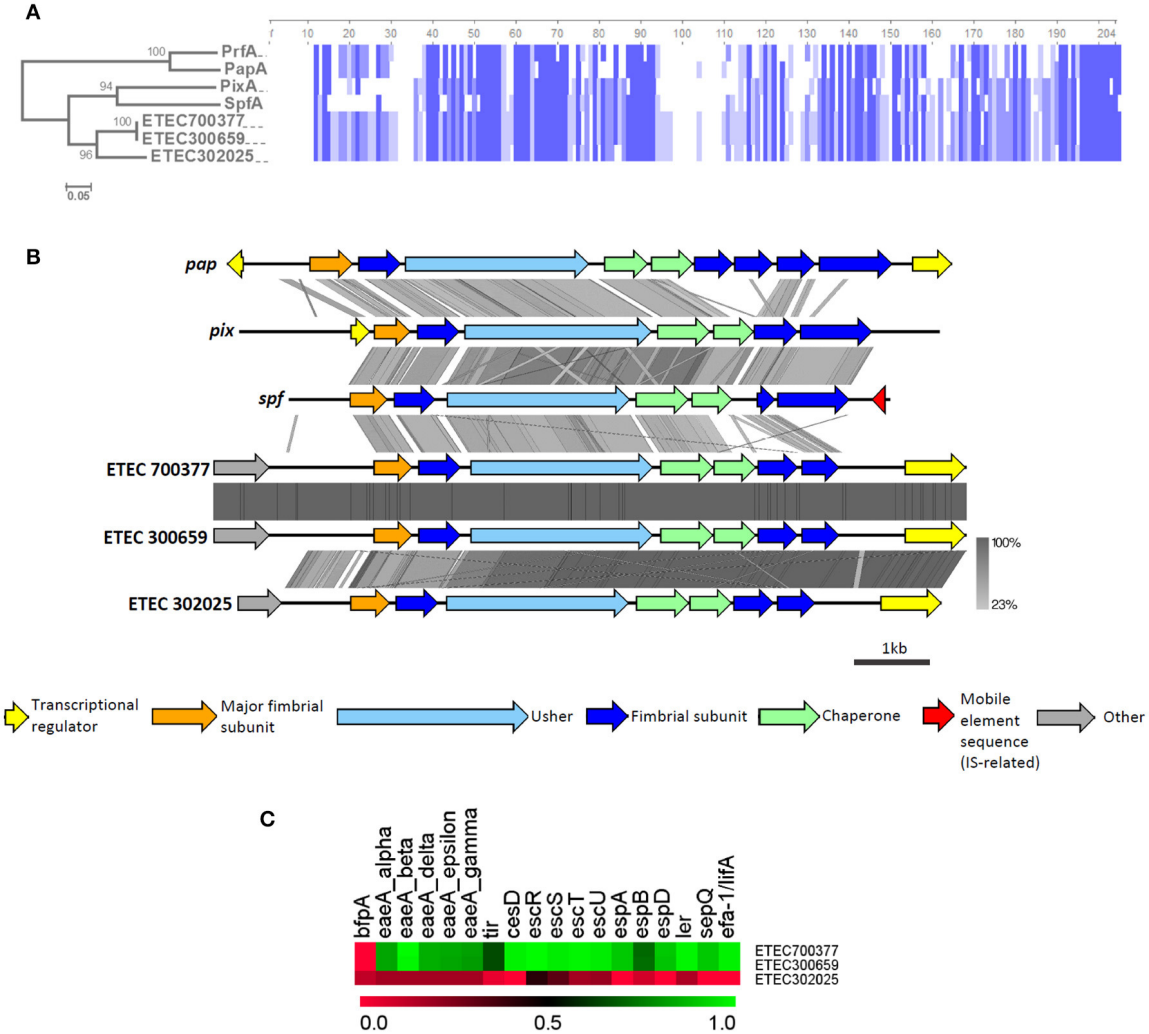
**Analysis of π-CU pili loci found in AN-ETEC strains**. **(A)** Alignment of the amino acid sequences of putative π-CU pili major structural subunits of AN-ETEC strains with those of known π-CU pili (PrfA, SpfA, PapA, PixA, and SpfA). Blue blocks indicate identical regions and the color fades as the identity percentage decreases. The tree was inferred from alignment by the neighbor joining method with 1000 bootstrap replicates. **(B)** Alignment of the predicted π-CU pili loci found in AN-ETEC strains using tblastx in Easyfig. Known π-CU pili loci *prfA, papA, pixA*, and *spfA* were included. **(C)** Heat map derived from the screening of EPEC virulence genes in genomes of AN-ETEC strains harboring π-CU pili loci.

None of the known ETEC CFs exhibit π-CU pili, but in our screening of usher genes, we identified three strains from the B_1_ phylogroup that were positive for homologs of the π-CU pili family (BSR values ranging between 0.70 and 0.82, Figure 4B), namely *sfpC, pixC, prfC*, and *papC*. These usher genes represent loci that differ from the π-CU pili loci *yfc, ybg*, and *yqi*, which were found to be widely distributed among *E. coli* strains (Wurpel et al., 2013). Two of these strains, ETEC 700377 and ETEC 300659, were closely related and were classified as ST-328, while the third, ETEC 302025, was classified as ST-155. Localization of the loci in these three genomes allowed for identification of clusters composed of seven ORFs that encoded four structural subunits, two chaperones and the usher. Alignment of the amino acid sequences derived from the PMS gene using known π-major structural subunits PapA, PrfA, PixA, and SpfA revealed a higher degree of identity with SpfA (67% identity with π-PMS of ETEC 700377 and ETEC 300659, and 65% with π-PMS of ETEC 302025; Figure 7A). Four stretches of four amino acids in the N-terminal region of the predicted mature proteins (Brunder et al., 2001), and one of seven amino acids in the C-terminal region found to be conserved across all the sequences included in the analysis (Figure 7A). Aligning the genetic clusters using tblastx showed similarities to known loci residing mainly in genes encoding the PMS, the adjacent gene encoding a putative structural subunit gene, the usher, and the two chaperones, although differing in the downstream putative structural subunit genes. In contrast, among the AN-ETEC π-CU pili loci (ETEC 700377/ETEC 300659 v/s ETEC 302025), dissimilarity resided mainly in the PMS and the adjacent putative subunit gene (Figure 7B).

A recently described hybrid enteropathogenic *E. coli* (EPEC)/ETEC strain harbors a π-CU pili locus, identical to the locus harbored by ETEC 700377 and ETEC 300659 (Dutta et al., 2015). Therefore, we computationally screened a set of EPEC genes in the three AN-ETEC strains in which we found π-CU pili loci; these genes were typically contained in the locus of enterocyte effacement (LEE), a pathogenicity island carried by both EPEC and enterohemorrhagic *E. coli* (Croxen et al., 2013). According to LS-BSR screening using tblastn, almost all of the EPEC genes are present in ETEC 700377 and ETEC 300659, except for *bfp* (Figure 7C). In contrast, none of the EPEC genes were found in ETEC 302025, suggesting that the presence of π-CU pili loci is not restricted to EPEC/ETEC hybrids.

### Beta CU family

To our knowledge, CU pili belonging to the β family have never been characterized, and the denomination as an actual pili family has been only been supported by sequence data (Nuccio and Bäumler, 2007). Although the presence of a β-CU pili locus in *E. coli* K-12 was reported, it is disrupted by an insertion sequence (IS), which may be why it has not received more attention (Korea et al., 2010). Our usher gene screening shows that 15 AN-ETEC genomes, in addition to the three *E. coli* K-12 substrains, also carry β-CU pili loci (BSR values ranging between 0.90 and 1.00, Figure 4B). All these strains belong to the phylogroup A. Based on the structure of the locus in *E. coli* K-12 substrain MG1655, 13 AN-ETEC genomes carry a complete cluster, lacking the IS and including five genes: *gltF*, a putative structural subunit; *yhcA*, a putative chaperone; *yhcD*, the putative usher; *yhcE*, whose function is unclear; and *yhcF*, another putative structural subunit (Nuccio and Bäumler, 2007; Korea et al., 2010). In one of the AN-ETEC strains (ETEC 401900) a complete locus was found, but a premature stop codon disrupts the *yhcD* gene. For this reason, this strain was not considered in further analyses.

**Figure 8:**
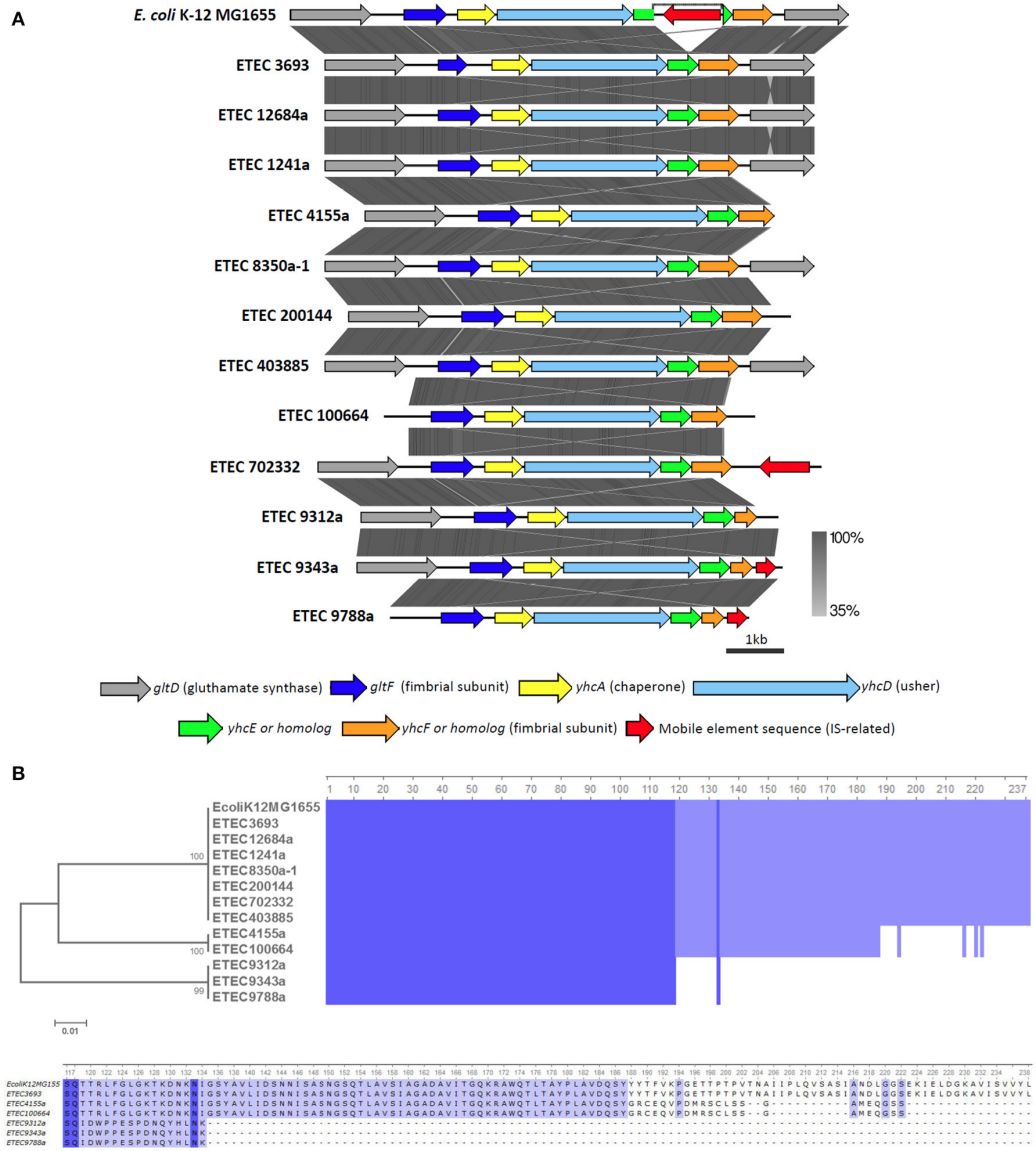
**Analysis of β-CU pili loci found in AN-ETEC strains**. **(A)** Alignment of the predicted β-CU pili loci found in AN-ETEC strains using tblastx in Easyfig. The known β-CU locus of *E. coli* K-12 MG1655 was included. **(B)** Alignment of the YhcE amino acid sequences found in AN-ETEC strains. YhcE of *E. coli* K-12 MG1655 was included. Blue blocks indicate identical regions and the color fades as the identity percentage decreases. The tree was inferred from alignment using the neighbor joining method with 1000 bootstrap replicates. The lower panel shows a zoom in the variable C-terminal region of YhcE.

All of the β-CU pili loci identified share the same organization, with the exception that the insertion sequence disrupting *yhcE* in *E. coli* K-12 is absent in all AN-ETEC genomes (Figure 8A). According to the alignment performed with tblastx, the *gltF, yhcA, yhcD*, and *yhcF* sequences are highly conserved among *E. coli* K-12 and AN-ETEC strains (Figure 8A). Only in the ETEC 963a-1 strain, in which sequencing did not allow for the identification of a complete β-CU pili locus, a longer form of *yhcE* was found. This variant likely encodes a protein with additional 57 amino acids in the C-term, unlike the form found in the other AN-ETEC strains. The variability among the identified β-CU pili loci can be almost exclusively attributed to *yhcF*, which was found in three different forms. One of them, of 717 bp, was found in nine AN-ETEC strains and corresponds to the form present in *E. coli* K-12. Two other shorter forms, of 633 and 405 bp, were identified in two and three AN-ETEC genomes, respectively (Figure 8A). Alignment of the predicted protein products is consistent with such an observation (Figure 8B). The three forms have an identical block of 118 amino acids and the divergence is determined by the length of the C-terminal regions.

In summary, at least one pili locus, different from those that seem to be widely distributed among *E. coli*, was identified in 33 of the 35 AN-ETEC strains included in this study. No loci were identified in strains ETEC 9571a (ONT, phylogroup A, ST-731, LT) and ETEC 603936 (O64, phylogroup B_1_, ST-155, STp-LT). We identified 10 different CU pili loci among the 35 AN-ETEC strains analyzed, which, according to the number of ORFs they contain, could encode functional pili structures. A list of these loci, along with their organization and positive strains, is provided in Table 2. None of the AN-ETEC strains seems to bear two β-, γ_2_-, κ-, or π-CU pili loci simultaneously, but they do harbor representatives of different families. In both, ETEC 100664 and ETEC 702251, β-, γ_2_-, and κ-CU pili loci were identified; while seven strains carry β-, and γ_2_-CU pili loci simultaneously (ETEC 8350a, ETEC 200144, ETEC 702332, ETEC 4155a, ETEC 9312a, ETEC 9343a, and ETEC 9788a). No β-, γ_2_-, nor κ-CU pili loci were identified among the AN-ETEC strains carrying π-CU pili loci.

**Table 2:** **Summary of the pili loci, or genes, identified in this work**.

**Locus (CU Family)**		**Locus organization**	**AN-ETEC strains**	**References**
*yhc* (β)	*gltFyhcADF*	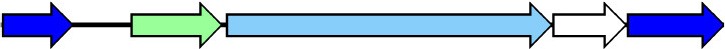	3693, 12684a, 1241a, 8350a-1, 200144, 702332, 403885	Korea et al., 2010
*yhc* (β)	*gltFyhcAD**F**_2_*	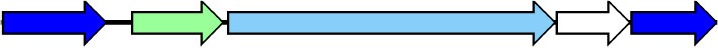	4155a, 100664	Korea et al., 2010; This work
*yhc* (β)	*gltFyhcAD**F**_3_*	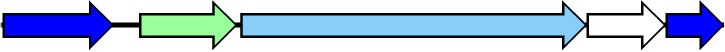	9312a, 9343a, 9788a	Korea et al., 2010; This work
*crs/CS26b* (γ_2_)	*crs**H**_*b*_BCDEFG*	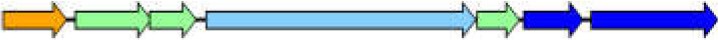	100664	Nada et al., 2011; This work
*cma/CS27b* (γ_2_)	*cma**H**_*b*_BCDEFG*	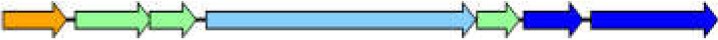	4155a, 603388, 1030c-1, 963a-1, 703200, 3693, 503210	Nada et al., 2011; This work
*cnm/CS28a* (γ_2_)	*cnm**H**_*a*_BCDEFG*	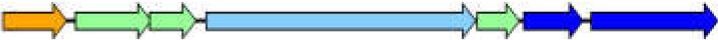	8350a-1, 200144, 702251	Nada et al., 2011; This work
*cnm/CS28b* (γ_2_)	*cnm**H**_*b*_BCDEFG*	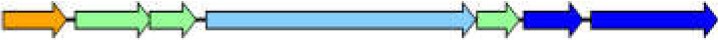	200617, 505077, 8255a	Nada et al., 2011; This work
*ctg* (γ_2_)	*ctgABCDEFG*	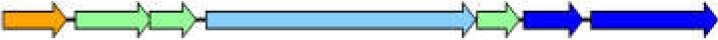	9312a, 9343a, 9788a, 401900	This work
*ctg* (γ_2_)	*ctg**A**_2_BCDEFG*	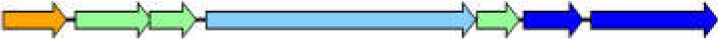	10380a	This work
*gep* (γ_2_)	*gepABCDEFG*	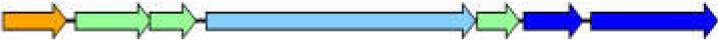	15617a, 203518	This work
*gte* (γ_2_)	*gteABCDEFG*	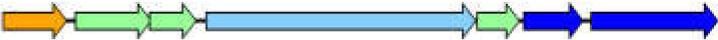	1241a	This work
*gtt* (γ_2_)	*gttABCDEFG*	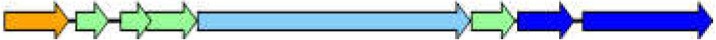	702332	This work
*aal* (κ)	*aalABCD***E**_2_*FGH*	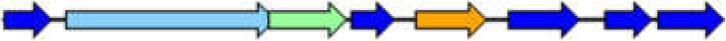	402594	Del Canto et al., 2012; This work
*aal* (κ)	*aalABCD**E**_3_FGH*	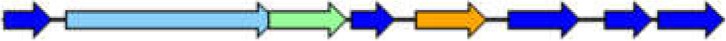	100664, 702251	Del Canto et al., 2012; This work
*ctp* (π)	*ctpAHCDEJF*	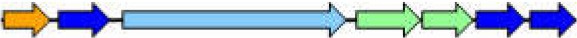	700377, 300659	Dutta et al., 2015; This work
*ctp* (π)	*ctp**A**_2_**H**_2_CDEJF*	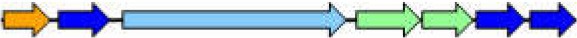	302025	This work

*
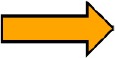
Major structural subunit 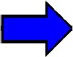
Structural subunit 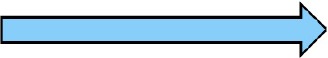
Usher 
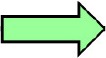
Chaperone 
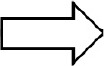
Unknown*.

*Genetic variants are in bold letters*.

In the case of previously described loci, we maintained the established names; for novel gene variants, these names were complemented with subscripts numbers or letters. We now propose five novel loci names. Thus, the locus name *yhc* was maintained for identified β-CU pili loci, with novel variants of *yhcF* being named *yhcF*_2_ and *yhcF*_3_ (Korea et al., 2010). For the γ_2_-CU pili loci, the previous names *crs/CS26, cma/CS27b, cnm/CS28a*, and *cnm/CS28b* were kept in the case of loci found in 14 AN-ETEC strains; and a novel variant of *crs/CS26* was identified (*crs/CS26b*) (Nada et al., 2011). Four novel names were given to the γ_2_-CU pili loci found in nine strains: *ctg* (CU ETEC gamma-2 pilus locus), *gep* (gamma-2 ETEC pilus locus), *gte* (gamma-two pilus locus of ETEC) and *gtt* (gamma-two pilus locus of toxigenic *E. coli*). As κ-CU pili loci were found in three AN-ETEC strains and displayed high identity with the *aal* locus along their entire extension, the name *aal* was maintained and variants for *aalE* were introduced (*aalE*_2_ and *aalE*_3_; Del Canto et al., 2012). Finally, the acronym *ctp* (CU ETEC pi pilus locus) was chosen for the π-CU pili loci found in three AN-ETEC strains, with variants for the putative structural subunit genes *ctpA* and *ctpH* (*ctpA*_2_ and *ctpH*_2_).

## Discussion

Understanding the infectious mechanisms of ETEC have been hampered by the fact that a significant portion of the ETEC isolates obtained from diarrhea cases worldwide have tested negative for currently known adhesins. Searching for and identifying novel adherence determinants can therefore contribute to the development of improved diagnostic methods and aid in the identification of potential targets for vaccines development and/or anti-adherence therapies. Massive sequencing and comparative genomics are valuable tools for these purposes (Sjöling et al., 2015), allowing the characterization of novel ETEC adhesin loci. We focused primarily on CU pili, as these are the main structures of currently known ETEC adhesins, particularly among CFs (Madhavan and Sakellaris, 2015). We chose a set of strains from different geographical locations and belonging to different serogroups. Enterotoxin profiles were not a requisite for strain selection, but most of the AN-ETEC strains were indeed positive for LT and not for ST. This is consistent with features of previously reported CF negative ETEC strains (Wolf, 1997; Shaheen et al., 2009; Rivera et al., 2010; Del Canto et al., 2011). Also consistent with previous evidence, most of our AN-ETEC strain collection belonged to the A and B_1_ phylogroups of *E. coli* (von Mentzer et al., 2014). This has been generally observed for ETEC strains, regardless of their virulence repertoire (von Mentzer et al., 2014). On the other hand, consistent with the diverse nature of ETEC strains, there were no predominant sequence types (von Mentzer et al., 2014).

As part of our goal, we aimed to find loci that were absent in non-pathogenic *E. coli*. Following this strategy, we identified 10 pili loci, distributed among 33 of the 35 AN-ETEC strains analyzed. Searching for genes encoding other adhesin types such as type IV pili, or proteins involved in their assembly, could allow the identification of additional loci, especially for cases such as the two strains resulting negative in our screening. In addition, these two strains may harbor CU pili homologous to those produced by non-pathogenic *E. coli*, a set that was excluded in our work. In this scenario, a detailed “case by case” analysis may be required to identify novel CU pili from alignment-based strategies, particularly when protein sequence identity fall to the 20–30% range, also known as the twilight zone (Blake and Cohen, 2001).

Representatives of the CU-pili families β, γ_2_, κ, and π, were found and described. The most common CU-CFs worldwide belong to the α (CFA/I, CS1, CS2, CS4, CS5, CS14, CS17, and CS19) and γ_3_ families (CS3 and CS6); these are the same CU-CFs that have been included in vaccine candidate formulations (O'Ryan et al., 2015; Zhang and Sack, 2015). CFs of the CU-families γ_2_ and κ are not the most common in ETEC strains (Isidean et al., 2011). However, the identification of several γ_2_-CU pili loci, described here and in previous works, suggests that they may be a numerous and important group among the ETEC adhesins. Certainly, wide screenings in ETEC collections are required to test this hypothesis. A search in databases using blastp, by introducing γ_2_, κ, and π-PMS described here, identified a limited number of identical registries. CrsH_b_ (γ_2_, one hit), CtgA (γ_2_, three hits), GepA (γ_2_, three hits), GteA (γ_2_, eight hits), and CtpA (π, three hits) were the only matched amino acid sequences with 100% identity and 100% coverage. In contrast, YhcE (β family) matched 353 registries, suggesting the absence of the insertion sequence that disrupts the *yhc* locus in *E. coli* K-12 strains. Upcoming massive sequencing projects and/or specific PCR screening will help to determine the global distribution of the loci identified here.

As sequencing projects are generating data to be deposited in databanks, assigning names to novel loci or proteins is not a trivial matter. We only assigned a name to a locus when, according to the number of genes it contains, it could direct the assembly of a potentially functional CU-pilus. We decided to keep the previously given names in the cases of the β-, κ-, as well as some of the γ_2_-CU pili loci identified in this work, as these showed high percentages of identity with previously described loci (>95%). When some of the genes identified showed more dissimilarity to their known homologs, but the rest of the locus was identical, we still used the original names from the literature, using numbers to differentiate the novel variants. Novel acronyms were introduced for five of the γ_2_- and the two of the π-CU pili loci. We have decided not to add novel representatives to the CF list until evidence of the existence of the pili has been confirmed and evaluation of their functional activity has been performed.

None of the CFs described to date belongs to the π family. Dutta et al. reported the presence of a homolog locus of *pap*, which encodes the P fimbria, in an ETEC strain that also has genes that used to be part of the LEE locus in atypical EPEC strains (Dutta et al., 2015). In the current project, we identified two π-CU pili loci, one of them identical to that described by Dutta et al. Our analysis was consistent in terms of the relatedness with the *pap* locus, but we found a higher identity with the *sfp* locus, which was first described in an enterohemorrhagic *E. coli* O157:H- strain, as is determinant in the capacity of agglutinating erythrocytes. These findings suggest that π-CU pili loci could also be part of the wide spectrum of ETEC adhesins. We estimated that percentages of identity to both the *pap* and *sfp* loci were not high enough to use one of these names for the novel ETEC π-CU pili loci. Therefore, we suggested the acronym *ctp*.

Among all the CU-pili families described in *E. coli* isolates, the β family is the one that has received the least attention. There are no published data on the production of β-CU pili or adhesin activity (Nuccio and Bäumler, 2007). In *E. coli* K-12 it is considered to be cryptic, because of the presence of an insertion sequence that would logically disrupt its functionality (Korea et al., 2010). However, the identification of non-disrupted β-CU pili loci in several AN-ETEC genomes, suggests that they may direct the assembly of functional structures. Two putative fimbrial subunits (GltF and YhcF), one chaperone (YhcA), and the usher (YhcD) are potentially encoded by β-CU pili loci, in addition to a protein with no predictable function (YhcE). A comprehensive review has suggested that, due to the lack of putative tip subunits, β-CU pili would be afimbrial structures or thin fibers (Nuccio and Bäumler, 2007).

Further research will be needed to prove the functional role of the CU pili loci identified in this work in ETEC pathogenesis. Preliminarily, we have obtained deletion mutants for a few representative ETEC strains and evaluated their capacity to attach to confluent monolayers of Caco-2 cells (Figure S2 in supporting information). ETEC strains 100664 and 702332 lacking γ_2_-CU loci *crs* and *gtt*, respectively, showed a reduced adhesion capacity compared to the wild type strains, even when this difference was only statistically significant in the case of *crs* (Figure S2). The same effect was noticed in ETEC strains 402594 and 302025 lacking the κ-CU locus *aal* (harboring *aalE*_2_ variant) and π-CU locus *ctp* (*ctpA*_2_*H*_2_ variant). No differences were observed in mutants lacking β-CU locus *yhc*, γ_2_-CU locus *cnm*, and π-CU locus *ctp*, compared to the wild types; while a significant increase was noticed in ETEC 9343a lacking the γ_2_-CU locus *ctg* (Figure S2). These results suggest that some of the identified CU pili loci encode determinants of *in vitro* bacterial adherence. Testing of the proper expression conditions and also other cell lines or adhesion substrates, may be determinant for future functional evaluations of the CU pili identified here. In addition, as other kind of structures different from CU pili may be directing AN-ETEC adherence to Caco-2 cells, double mutants or recombinant expression in non-pathogenic *E. coli* strains could be required.

In conclusion, ETEC strains lacking currently known CFs bear genes that encode related structures, particularly CU-pili of the γ_2_ and κ families. Beta- and π-CU pili are new members within the diverse ETEC adhesin repertoire. These findings add novel and valuable data to the large list of potential ETEC “weapons.” Future investigation will characterize their specific role in pathogenicity and whether they are suitable antigens for vaccine candidate formulations.

## Author contributions

FD: study design, strain characterization, data analysis, bioinformatic analysis, manuscript writing. MO: study design, data analysis, manuscript writing. MP: strain characterization, data analysis. AT: strain characterization, data analysis. DG: stain characterization, data analysis. RV: bioinformatic analysis. LC: bioinformatic analysis. AM: strain characterization, data analysis. RM: strain characterization, data analysis. DH: strain characterization, data analysis. BC: strain characterization, data analysis. MML: study design, data analysis. CMH: data analysis, bioinformatic analysis. MP: data analysis, bioinformatic analysis. DAR: data analysis, bioinformatic analysis, manuscript writing. OCS: study design, data analysis, bioinformatic analysis, manuscript writing. RV: study design, data analysis, manuscript writing.

## Funding

This work was funded with the support of the following grants: Fondo Nacional de Desarrollo Científico y Tecnológico (FONDECYT) grants 3130555 (FD), 11150966 (FD), 1110260 (RV), 1161161 (RV). Grant ID 38874, “Diarrheal disease in infants and young children in developing countries” and Grant ID 1016839 “Metagenomics Based Discovery of New Viral and Eukaryotic Pathogens Causing Diarrheal Disease” from the Bill and Melinda Gates Foundation.

### Conflict of interest statement

The authors declare that the research was conducted in the absence of any commercial or financial relationships that could be construed as a potential conflict of interest.

## Supplementary material

Figure S1 **(A)** Alignment of nucleotide sequences of the gene encoding a putative type-IV pilin, found in ETEC 401909, with genes cofA and lngA encoding major subunits of CS8 and CS21, respectively. The internal regions shown correspond to those recognized by primers (forward, left; reverse, right) used in CFs detection. **(B)** Alignment of amino acid sequences of the pilins of CS8, CS21, CS8b, and that found in ETEC 401909.

Figure S2 **Evaluation of the adherence capacity of ETEC strains to Caco-2 cells, after knocking out CU pili loci identified in this work. (A)** β-CU: Locus *yhc* in ETEC 100664, **(B)** γ_2_-CU: locus *crs* in ETEC 100664, locus *cnm* in ETEC 8350a-1, locus *crs* in ETEC 9343a, and locus *gtt* in ETEC 702332 (all loci belonging to family), **(C)** κ-CU: locus *aal* in ETEC 402594, **(D)** π-CU: locus *ctp* in ETEC 300659, and locus *ctp* in ETEC 302025. The graphics show the number (average ± *SEM*) of cell-associated colony forming units (CFU) recovered after 3 h of infection. Mutant strains were obtained by allelic exchange mediated by the lambda red recombinase system using pKD46 or pSIMs plasmids (Datsenko and Wanner, 2000; Sharan et al., 2009) and the primers listed in the table below. Adherence capacity was evaluated by infecting confluent Caco-2 monolayers with a multiplicity of 10 bacteria per cell, for 3 h at 37°C in a 5% CO_2_ atmosphere. After repeated washes with saline buffer, cells were lysed with 0.1% Triton X-100. The suspension was serially diluted and seeded onto LB agar plates for CFU counting. Data were analyzed using unpaired *t*-test with Welch's correction and differences were considered significant when *P* < 0.05.

Table S1 **Accession codes for gene sequences used in this work**.

Table S2 **PCR primers used for CF gene detection**.

Table S3 **Chromosome and plasmid sequences of ***Escherichia coli*** strains accessed in this work**.

Data Sheet 1 **Putative major subunits encoded by CU-pili loci identified in this work**.
