# Supplementary material for: Chaperone-Usher Pili Loci of Colonization Factor-Negative Human Enterotoxigenic Escherichia coli
Source: Front Cell Infect Microbiol. 2017 Jan 6;6:200. doi: 10.3389/fcimb.2016.00200 (PMC5216030; doi:10.3389/fcimb.2016.00200)
Supplement: Figure S1 — (A) Alignment of nucleotide sequences of the gene encoding a putative type-IV pilin, found in ETEC 401909, with genes cofA and lngA encoding major subunits of CS8 and CS21, respectively. The internal regions shown correspond to those recognized by primers (forward, left; reverse, right) used in CFs detection. (B) Alignment of amino acid sequences of the pilins of CS8, CS21, CS8b, and that found in ETEC 401909. [file Image1.PDF]

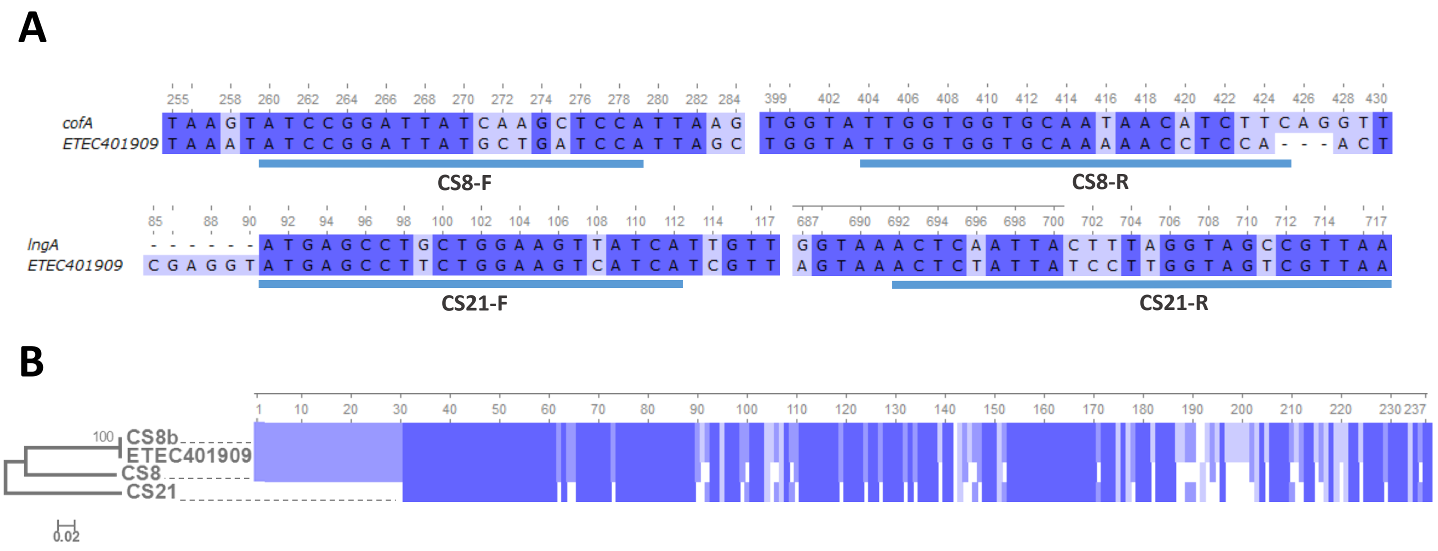

Figure S1. **A)** Alignment of nucleotide sequences of the gene encoding a putative type-IV pilin, found in ETEC 401909, with genes *cofA* and *lngA* encoding major subunits of CS8 and CS21, respectively. The internal regions shown correspond to those recognized by primers (forward, left; reverse, right) used in CFs detection. **B)** Alignment of amino acid sequences of the pilins of CS8, CS21, CS8b and that found in ETEC 401909.
