# Supplementary material for: Chaperone-Usher Pili Loci of Colonization Factor-Negative Human Enterotoxigenic Escherichia coli
Source: Front Cell Infect Microbiol. 2017 Jan 6;6:200. doi: 10.3389/fcimb.2016.00200 (PMC5216030; doi:10.3389/fcimb.2016.00200)
Supplement: Figure S2 — Evaluation of the adherence capacity of ETEC strains to Caco-2 cells, after knocking out CU pili loci identified in this work. (A) β-CU: Locus yhc in ETEC 100664, (B) γ2-CU: locus crs in ETEC 100664, locus cnm in ETEC 8350a-1, locus crs in ETEC 9343a, and locus gtt in ETEC 702332 (all loci belonging to family), (C) κ-CU: locus aal in ETEC 402594, (D) π-CU: locus ctp in ETEC 300659, and locus ctp in ETEC 302025. The graphics show the number (average ± SEM) of cell-associated colony forming units (CFU) recovered after 3 h of infection. Mutant strains were obtained by allelic exchange mediated by the lambda red recombinase system using pKD46 or pSIMs plasmids (Datsenko and Wanner, 2000; Sharan et al., 2009) and the primers listed in the table below. Adherence capacity was evaluated by infecting confluent Caco-2 monolayers with a multiplicity of 10 bacteria per cell, for 3 h at 37°C in a 5% CO2 atmosphere. After repeated washes with saline buffer, cells were lysed with 0.1% Triton X-100. The suspension was serially diluted and seeded onto LB agar plates for CFU counting. Data were analyzed using unpaired t-test with Welch's correction and differences were considered significant when P < 0.05. [file Image2.PDF]

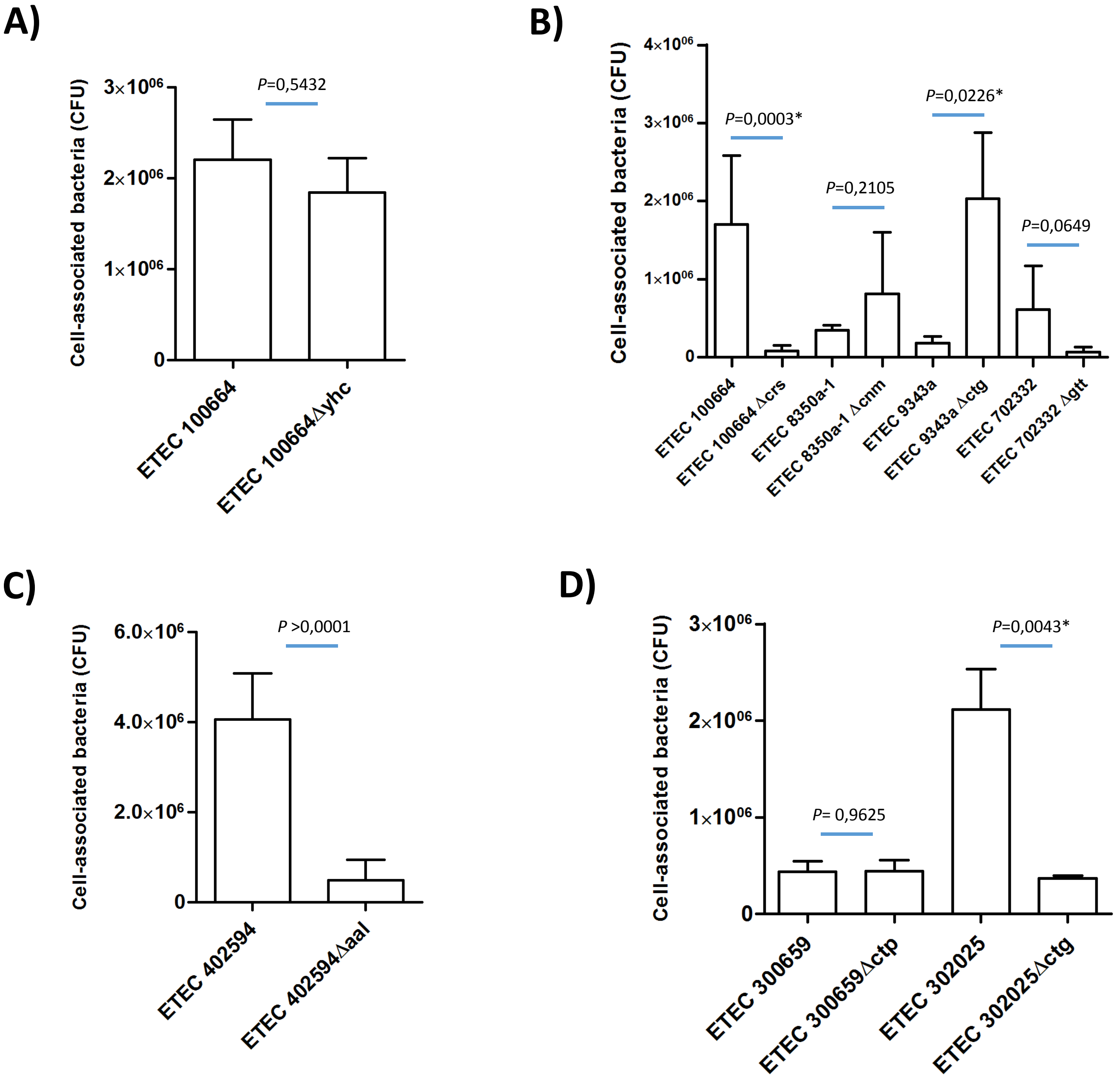

Figure S2. Evaluation of the adherence capacity of ETEC strains to Caco-2 cells, after knocking out CU pili loci identified in this work. **A)** β-CU: Locus *yhc* in ETEC 100664 **B)** γ_2-CU: Locus *crs* in ETEC 100664, locus *cnm* in ETEC 8350a-1, locus *crs* in ETEC 9343a, and locus *gtt* in ETEC 702332 (all loci belonging to family) **C)** κ-CU: Locus *aal* in ETEC 402594 **D)** π-CU: Locus *ctp* in ETEC 300659, and locus *ctp* in ETEC 302025. The graphics show the number (average ± SEM) of cell-associated colony forming units (CFU) recovered after 3h of infection. Mutant strains were obtained by allelic exchange mediated by the lambda red recombinase system using pKD46 or pSIMs plasmids (Datsenko and Wanner, 2000; Sharan *et al*, 2009) and the primers listed in the table below. Adherence capacity was evaluated by infecting confluent Caco-2 monolayers with a multiplicity of 10 bacteria per cell, for 3h at 37 °C in a 5% CO_2 atmosphere. After repeated washes with saline buffer, cells were lysed with 0,1% Triton X-100. The suspension was serially diluted and seeded onto LB agar plates for CFU counting. Data were analyzed using unpaired t test with Welch's correction and differences were considered significant when P<0,05.

Primers used for mutagenesis of CU-pili loci

Primer	Sequence (5' to 3')	Use	Reference
Beta-CmF	AAGGATATGTTCATATGTTTTTCAAAAAGAACCTCACAACGTGTAGGCT GGAGCTGCTTC	Mutagenesis (β-*yhc* locus)	This work
Beta-CmR	CTAATTGATGCTGTTCCTGTGCTGCCCGGATTGTCATTTTCATATGAATA TCCTCCTTAG	Mutagenesis (β-*yhc* locus)	This work
Beta-F	CAATCCGTCTCAATTACTTGTTAAC	Mutagenesis confirmation (β-*yhc* locus, paired with Cm-F)	This work
G2c-CmF	ATGAAAAAAGACAATTATGTCTCTGGCTGTGGTTTCAGCTTGTGTAGGCT GGAGCTGCTTC	Mutagenesis (γ_2-*crs* locus)	This work
G2c-CmR	CTAGTTCTCCCATCGGGAGATTATTTTTATTGATACATTACATATGAATA TCCTCCTTAG	Mutagenesis (γ_2-*crs* locus)	This work
G2d-F	AGGTCGAACACTTAACAATTAGG	Mutagenesis confirmation (γ_2-*crs* locus, paried with Cm-F)	This work
G2a-CmF	GAGGTTTTGTAATGAAGAAAACAATTATGTTTTTTGCGGTGTGTAGGCT GGAGCTGCTTC	Mutagenesis (γ_2-*ctg* locus)	This work
G2a-CmR	TACAGCTTTCTGGCCAATTACTGATGGCACACCAAACTTCATATGAATAT CCTCCTTAG	Mutagenesis (γ_2-*ctg* locus)	This work
G2a-F	GCCTTTGAGGTCGTGGTATG	Mutagenesis confirmation (γ_2-*ctg* locus, paried with Cm-F)	This work
G2b-CmF	ATGAAAAAAATGATTATGCCTTTAACTATGGTGTCCGTTCGTGTAGGCT GGAGCTGCTTC	Mutagenesis (γ_2-*cnm* locus)	This work
G2b-CmR	TCAGGAATCGCTCCATCTCGAAACAATAGTAAGTGGAATGCATATGAAT ATCCTCCTTAG	Mutagenesis (γ_2-*cnm* locus)	This work
G2b-F	GCTAAACCCATAAGCTAGACTC	Mutagenesis confirmation (γ_2-*cnm* locus, paried with Cm-F)	This work
G2e-CmF	ATGAACATGAAAAGAAATTTGTTAAGTTTATCGTTCTTGGGTGTAGGCT GGAGCTGCTTC	Mutagenesis (γ_2-*cgtt* locus)	This work
G2e-CmR	TTAAGATCGTTCCCATCTCGTTACTATATTTAAAGCAACACATATGAATA TCCTCCTTAG	Mutagenesis (γ_2-*cgtt* locus)	This work
G2e-F	AATCGGTGGAGCTGCATGAC	Mutagenesis confirmation (γ_2-*gtt* locus, paried with Cm-F)	This work
Ckt-CmF	GAGATGACGAACACCGGACTGCGGGTGAGCTGGTCACGGCGTGTAGG CTGGAGCTGCTTC	Mutagenesis (κ-*aal* locus)	This work
Ckt-CmR	TCACTACCCTCAAAAGCACGGGCTGATTTACCGGCAAGCACATATGAAT ATCCTCCTTAG	Mutagenesis (κ-*aal* locus)	This work
Ckt check-F	CGATGACAGTGACCTGAAAG	Mutagenesis confirmation (κ-*aal* locus, paried with Cm-F)	This work
Pi-CmF	TATTTTACCGTTAATAACATTACCTATGCGTTGATGCTTTGTGTAGGCTG GAGCTGCTTC	Mutagenesis (π-*ctp* locus)	This work
Pi-CmR	TTATATATAAAAAATATTCATCGTAACTGTCGCACTAAATCATATGAATA TCCTCCTTAG	Mutagenesis (π-*ctp* locus)	This work
Pa-F	TGGTGGCTCTTTCGTTACTAAC	Mutagenesis confirmation (π-*ctp* locus, paried with Cm-F)	This work
Cm-R	ATGAAAGACGGTGAGCTGGT	Mutagenesis confirmation	Gutiérrez et al, 2016
K1	CAGTCATAGCCGAATAGCCT	Mutagenesis confirmation	Datsenko and Wanner, 2000

References

1. Datsenko, K.A., Wanner, B.L. (2000). One step inactivation of chromosomal genes in *Escherichia coli* K-12 using PCR products. *Proc. Natl. Acad. Sci. USA*. 97:6640–6645.
2. Sharan, S.K., Thomason, L.C., Kuznetsov, S.G., Court, D.L. (2009). Recombineering: a homologous recombination-based method of genetic engineering. Nat Protoc. 4:206-223. doi: 10.1038/nprot.2008.227.
3. Gutiérrez, D., Pardo, M., Montero, D., Oñate, A., Farfán, M.J., Ruiz-Pérez, *et al*. (2015). TleA, a Tsh-like autotransporter identified in a human enterotoxigenic *Escherichia coli* strain. *Infect. Immun*. 83:1893-903. doi: 10.1128/IAI.02976-14.
