# Supplementary material for: Chaperone-Usher Pili Loci of Colonization Factor-Negative Human Enterotoxigenic Escherichia coli
Source: Front Cell Infect Microbiol. 2017 Jan 6;6:200. doi: 10.3389/fcimb.2016.00200 (PMC5216030; doi:10.3389/fcimb.2016.00200)
Supplement: Table S2 — PCR primers used for CF gene detection. [file Table2.DOCX]

**Table S2**: PCR primers used for CF gene detection

| **CF** | **Primer** | **Sequence (5’-3’)** | **Product size (bp)** | **Reference** |
| --- | --- | --- | --- | --- |
| CFA/I | F | ACTATTGGTGCAATGGCTCTGAC | 497 | Vidal et al, 2009 |
|  | R | CAGGATCCCAAAGTCATTACAAG |  |  |
| CS1 | F | GAGAAGACCATTAGCGTTACGG | 410 | Vidal et al, 2009 |
|  | R | CCCTGATATTGACCAGCTGTTAG |  |  |
| CS2 | F | ACTGTAACTGCTAGCGTTGATCC | 358 | Vidal et al, 2009 |
|  | R | TGCTTCCTGCATTAATAACGAGT |  |  |
| CS3 | F | CCCACTCTAACCAAAGAACTGG | 300 | Vidal et al, 2009 |
|  | R | CGTATTTCCAGCATTTTTATCCA |  |  |
| CS4 | F | ATTGATATTTTGCAAGCTGATGG | 242 | Vidal et al, 2009 |
|  | R | GTCACATCTGCGGTTGATAGAGT |  |  |
| CS5 | F | CAACCGTATCAGGTTCTGTTTTG | 558 | Vidal et al, 2009 |
|  | R | CAAATGTTACCGGAGCTACAAAG |  |  |
| CS6 | F | AAATGTATCCCAGGTAACGGTCT | 165 | Vidal et al, 2009 |
|  | R | TGTTGATTAGGCGTAACCTCTGT |  |  |
| CS7 | F | TGCTCCCGTTACTAAAAATAC | 203 | Del Canto et al, 2011 |
|  | R | TAGATGTCGTATCACTACGT |  |  |
| CS8 | F | ATCCGGATTATCAAGCTCCA | 166 | Rodas et al, 2009 |
|  | R | GAAGATGTTATTGCACCACCAA |  |  |
| CS12 | F | GCGAATAACAATGATGCAAG | 263 | Del Canto et al, 2011 |
|  | R | CCTGACTGGTTTACAAGATA |  |  |
| CS13 | F | GGGACTGCCACAATGAATTT | 178 | Rodas et al, 2009 |
|  | R | CAGCACCACCTGCTGATTTA |  |  |
| CS14 | F | TTTGCAACCGACATCTACCA | 162 | Rodas et al, 2009 |
|  | R | CCGGATGTAGTTGCTCCAAT |  |  |
| CS15 | F | CGAAATTGGACAAGCGATG | 130 | Rodas et al, 2009 |
|  | R | TCCAGCAGGGATATTATTCG |  |  |
| CS17-19 | F | TAAACTTGATCTTCTGCAAGC | 324 | Del Canto et al, 2011 |
|  | R | GCATGAATCGTAAGCTGTTG |  |  |
| CS17 | R | TCAGGCGCAGTTCCTTGTGTG (paired with CS17-19F) | 348 | Del Canto et al, 2011 |
| CS18 | F | ATCCGTCAGGTGTTTGTGGT | 362 | Rodas et al, 2009 |
|  | R | CACCTGAATTCCTCGACAGG |  |  |
| CS20 | F | AGGTATCCAAATCCGCACTG | 114 | Rodas et al, 2009 |
|  | R | CATCAGCCAGCACATAGGAA |  |  |
| CS21 | F | TCATGAGCCTGCTGGAAGTTATCA | 617 | Rodas et al, 2009 |
|  | R | TCCGGCTACCTAAAGTAATTGAGT |  |  |
| CS22 | F | ATTGGACAAGCGTCCAACAC | 127 | Rodas et al, 2009 |
|  | R | TTCCAGCAGGGATATTATCATTTT |  |  |
| CS23 | F | CTGCTATGGCGTGGACTGTA | 597 | Del Canto et al, 2012 |
|  | R | AGGTGAATAGGGGGTTCTCG |  |  |

References for Table S2:

1. Vidal, R.M., Valenzuela, P., Baker, K., Lagos, R., Esparza, M., Livio, S., *et al*. (2009) New multiplex PCRs for characterization of enterotoxigenic *Escherichia coli* (ETEC) colonization factor antigen genes. *Diagn. Microbiol. Infect. Dis*. 65:217–223.
2. Del Canto, F., Valenzuela, P., Cantero, L., Bronstein, J., Blanco, J.E., Blanco, J., *et al*. (2011). Distribution of classical and nonclassical virulence genes in enterotoxigenic *Escherichia coli* isolates from Chilean children and tRNA gene screening for putative insertion sites for genomic islands. *J. Clin. Microbiol.* 49: 3198-3203.
3. Rodas, C, Iniguez, V., Qadri, F., Wiklund, G., Svennerholm, A.M., Sjöling, A. (2009). Development of multiplex PCR assays for detection of enterotoxigenic *Escherichia coli* colonization factors and toxins. *J. Clin. Microbiol.* 47:1218 –1220.
4. Del Canto, F., Botkin, D.J., Valenzuela, P., Popov, V., Ruiz-Perez, F., Nataro, J.P., *et al*. (2012). Identification of coli surface antigen 23, a novel adhesin of enterotoxigenic *Escherichia coli*. *Infect. Immun.* 2012; 80: 2791-2801.
