# Supplementary material for: Chaperone-Usher Pili Loci of Colonization Factor-Negative Human Enterotoxigenic Escherichia coli
Source: Front Cell Infect Microbiol. 2017 Jan 6;6:200. doi: 10.3389/fcimb.2016.00200 (PMC5216030; doi:10.3389/fcimb.2016.00200)
Supplement: Table S3 — Chromosome and plasmid sequences of Escherichia coli strains accessed in this work. [file Table3.docx]

| **Table S3. Chromosome and plasmid sequences of *Escherichia coli* strains accessed in this work** | | |
| --- | --- | --- |
| **Strain (Chromosome)** | **Plasmid** | **GenBank accession code** |
| *Escherichia coli* ATCC 8739 |  | NC_010468.1 |
| *Escherichia coli* B str, REL 606 |  | NC_012967.1 |
| *Escherichia coli* BL21(DE3) |  | NC_012971.2 |
| *Escherichia coli* ED1a |  | NC_011745.1 |
|  | pECOED | NC_011754.1 |
| *Escherichia coli* HS |  | NC_009800.1 |
| *Escherichia coli* IAI1 |  | NC_011741.1 |
| *Escherichia coli* str. K-12 substr. DH10B |  | NC_010473.1 |
| *Escherichia coli* str. K-12 substr. MG1655 |  | NC_000913.3 |
| *Escherichia coli* str. K-12 substr. W3110 |  | NC_007779.1 |
| *Escherichia coli* SE11 |  | NC_011415.1 |
|  | pSE11-1 | NC_011419.1 |
|  | pSE11-2 | NC_011413.1 |
|  | pSE11-3 | NC_011416.1 |
|  | pSE11-4 | NC_011407.1 |
|  | pSE11-5 | NC_011408.1 |
|  | pSE11-6 | NC_011411.1 |
| *Escherichia coli* SE15 |  | NC_013654.1 |
|  | pECSF1 | NC_013655.1 |
| *Escherichia coli* SMS-3-5 |  | NC_010498.1 |
|  | pSMS35_130 | NC_010488.1 |
|  | pSMS35_3, | NC_010487.1 |
|  | pSMS35_4 | NC_010486.1 |
|  | pSMS35_8 | NC_010485.1 |
| *Escherichia coli* W |  | NC_017635.1 |
|  | pRK1 | NC_017637.1 |
|  | pRK2 | NC_017636.1 |
| *Escherichia coli* E24377A |  | NC_009801.1 |
|  | pETEC_35 | NC_009787.1 |
|  | pETEC_5 | NC_009791.1 |
|  | pETEC_6 | NC_009789.1 |
|  | pETEC_73 | NC_009788.1 |
|  | pETEC_74 | NC_009790.1 |
|  | pETEC_80 | NC_009786.1 |
| *Escherichia coli* B7A |  | AAJT02000001-AAJT02000289 (whole genome shotgun) |
| *Escherichia coli* ETEC H10407 |  | NC_017633.1 |
|  | p52 | NC_017721.1 |
|  | p58 | NC_017723.1 |
|  | p666 | NC_017722.1 |
|  | p948 | NC_017724.1 |
