# Supplementary material for: Chaperone-Usher Pili Loci of Colonization Factor-Negative Human Enterotoxigenic Escherichia coli
Source: Front Cell Infect Microbiol. 2017 Jan 6;6:200. doi: 10.3389/fcimb.2016.00200 (PMC5216030; doi:10.3389/fcimb.2016.00200)
Supplement: Data Sheet 1 — Putative major subunits encoded by CU-pili loci identified in this work. [file DataSheet1.PDF]

Putative major subunits encoded by CU-pili loci identified in this work

For β-CU, the amino acid sequence of YhcE, absent in *E. coli* K-12, and three variants of YhcF are shown.

β-CU pili

YhcE
MKRIITGCLLLNFAMAAQAECNISSSIQNIDYGKRSAAMRQVDRGKTTQLADRTITLVMQCDQDAHIRVQLNT
ANISNNGFGFGPNGSLNLIASDAFSGSNNLDLALASGKNDNPGSTGTASISTSPNNWLVFMQNGQEVVIDSGK
SVSLTLTMAPAFKDEGELTDMTDITGNLTVLVEAK

YhcF
MNNVKLLIAGSAFFAMSAQAADRVSIDVKVTLEAAACTPILSNGGVVNFGSHSVNRLSTQHYTQIGTRNINMT
ITCESATGIAITARDTRMDSMTTGKDSGGQSGVKYTLNGGGYISQTTRLFGLGKTKDNKNIGSYAVLIDSNNI
SASNGSQTLAVSIAGADAVITGQKRAWQTLTAYPLAVDQSYYYTFVKPGETTPTPVTNAIIPLQVSASIAND
LGGSEKIELDGKAVISVVYL

YhcF$_2$
MNNVKLLIAGSAFFAMSAQAADRVSIDVKVTLEAAACTPILSNGGVVNFGSHSVNRLSTQHYTQIGTRNINMT
ITCESATGIAITARDTRMDSMTTGKDSGGQSGVKYTLNGGGYISQTTRLFGLGKTKDNKNIGSYAVLIDSNNI
SASNGSQTLAVSIAGADAVITGQKRAWQTLTAYPLAVDQSYGRCEQVPDMRSCLSSGAMEQGSS

YhcF$_3$
MNNVKLLIAGSAFFAMSAQAADRVSIDVKVTLEAAACTPILSNGGVVNFGSHSVNRLSTQHYTQIGTRNINMT
ITCESATGIAITARDTRMDSMTTGKDSGGQSGVKYTLNGGGYISQIDWPPESPDNQYHLNK

γ_2-CU pili

CrsH$_b$
MKKTIMSLAVVSALVSGVAFADPPKNDSSKATVNFNGKVTSSLCQVRTDNISQDISLGEVTTTVLQGSGKGKP
QSFEVGLTNCDTATNKISYVLSDANYTPIVNGGQATNLNYLVPKSGDNSAVGVGVYVETSTGTAIAPGSTTEL
KVEKDAENKALSNQTISLRAYIGTKTGTADPAAASQVEAGTVEATGVLTIYAI

CmaH$_b$
MKKTIMSLAVVSALVSGSAFAAAPQNDSSKATINFNGKVTSSLCQVRTDNLSQDISLGEVTATVLKDSGKGKA
QSFQVGLTNCDTTTSKISYVLTDGNYTPSLDSNNAAVKLSYLQPKSGDNSADGVGVFIETSTGTAIAPGSTTN
LDVAKDGKSQALSDQTISLRAYIGTKDGNPDANQSVKAGTVEATGILTIYAGV

CnmH$_a$
MKKMIMPLTMVSVLMSGSALAKPGANDSSQATLNFNGRVTSSLCQVKTDDVVKDIYLGEVSKSALEANAQGPK
QSFQVNLINCDTTVSDISYVLSDANGNGTAAYLIPKSGDTSATGVGVYVEKSDGTSVGVGDTQTLTVTKNDAN
ALSEQVIPLRAYIRAQGGAGGVTAGTVDATGILTIRATANP

CnmH$_b$
MKKMIMPLTMVSVLMSGSALAAPGANDSSQATLNFNGRVTSSLCQVKTDDVVKDIYLGEVSKSALEANAQGPK
QSFQVNLINCDTTVNDISYVLSDANGNGTANYLIPKSGDTSATGVGVYVEKSDGTPVNVGNTQTLTVTKKDGT
DALSEQVIPLRAYIGAQGGAGGVSAVTAGTVDATGILTIRATKP

CtgA
MKKTIMFFAVASALFSGAAMAAPAANDASTATLNFTGRVTSSLCQVDTSSVAQNISLGEVSASALKATGVAPA
KSFSVSLVNCDSGTGKITYNIADANASGANPTADYLQPLSEDDSARGVGVFIQSSDHQDITIGQDKELAVVKD
QSGSALSKQTISLSAYMATTDKAADTTGSKVSPGSVSARGVMTIKAAN

$CtgA_2$
MKKTIMFFAVASALFSGAVMAAPAANNASTATLDFTGRVTSSLCQVDTSSVAQNISLGEVSASALKATGIAPA
KSFSVSLVNCDSETGKITYNIADANASGAGATAAYLQPLSEDDSARGVGVFIQSSDHQDITIGQDKDLAVVKD
QSGSALSKQTISLSAYMATTDKTADANGSKVTPGSVSARGVMTIKAATTN

GepA
MKKTIMFFAVVSALFSAGAMAANSVNANGQADNSASTATLNFTGKVTSSLCQVNTSDLTKTISLGEVSQAVLN
TDGAHSPSQSFTVGLSNCDPTVSNITYVIRDGNASPATGVNTSAYLIPNSGDTTASGVGVYVAEPEGRAIQIG
ENKNLSVVKDGQGQALSEQTISLAAYIKKVGAGAVTAGTVDAQGIMTIKATATGG

Gte
MKKTIMSFAVVSALFSGAAMAALTNGQADNSASSATLNFSGKVTSSLCQVSTSALTKTISLGEASAAALANGG
QSPFQSFTVSLANCDPTTTKITYTIRDGNGLPGQDAHTSDYLIPKSGDTSASGVGVYIADPEHKAIQIGANQN
AGVTQVGGQAESEQTIALTAYMKATGNAGNVKAGVVDATGIMTIKAATASVGG

Gtt
MNMKRNLLSLSFLAFFGVSGTALAAASAPAQNDSSTANLEFTGKVTSSLCQVSTSDLNQSIKLGEVSATALGN
GGKSPAQSFTVTLNNCATNTGTINYVFSDTNGSPGTTSYLVPLAGDTSASGVGVYLEKSNGTAITIGQTNNID
VTKGADGTSALPQQSIPLKAYIGKINSSATVIPGDVTANAVMTIRTVESASP

κ-CU pili

$AalE_2$
MKYQGIYFMKKTLIALAVAASAAVSGSAMAWTANGTGGSVELGGTVNVLSPNSPWEVKVGDAVNNLNAEIHSG
DKKVVIPANREIPILGIRTVSNVPFHGQNGISPQIDFGKAVNIDSFQNGRADLTLDVKNDSDVKIGTLTTIFT
SGAEGSTSNGQTSSKFNLFAANPGDAFYGGLGKSNDKISNEGWYIAHLFGAEYVQNYNDQNGTFVAEGNHEFF
NNSQFTYSAVYAAGILDNANITITLEQPATSNITWKASLPVSVTYQ

$AalE_3$
MKYQGIYFMKKTLIALAVATSAAVSGSAMAWTANGDGGSVSLSGTVNVLSPNTPWEVKVGDAVNNL
NLEIHSGDREAAIPVQKIIPILGIRTASHDPFHGQTGISPQIDFGNAINTDSFEGGRTGLTLEVKN
DSDVKIGTLTTILTSGAEASAAVGNTRSKFNLFAANQGHAFYGGLGKSNDKISQNSWYIANLFGND
YVENYNDQGGNLVASGSYQYFNDPQITYSAVYAAGILNNTDTNITITLDQPATSNITWKASLPIRV
TYQ

π-CU pili

CtpA
MKKSLVAGVVAMAMFSFGAQANGQGVVNFKGSVIDAPCGIASESADQTIDFGQISKAHLAAAGISVKKDLDIK
LVNCSLAKPGSNPAASFKTVKVAFTGSTVGGQADELGTTGNTGTAIVVSEAGGKLVKFNGTAGDTSNLQDGDT
TLRYSAWVKKATNGTLKEGDFAAVANFNLTYQ

$CtpA_2$
MKKSLVAGVVAMAMFSFGAQANGGQGVVNFKGSVIDAPCGIASGSADQTIDFGQISKAALEANGTSVKQNLDI
KLVNCSLAKAPAAGGAAQAPFKRVTVSFTGTTINGQNNELGTTGKTGTAIVVSDASGKSVDFTGTAGAASNLQ
DGDNTLRYSAWVKKATTGTLSEGDFSAVANFNLTYQ
